# Epsilon poly-L-lysine as a novel antifungal agent for sustainable wood protection

**DOI:** 10.3389/fmicb.2022.908541

**Published:** 2022-09-07

**Authors:** Lili Cai, Chi-Jui Kuo

**Affiliations:** Department of Forest, Rangeland and Fire Sciences, University of Idaho, Moscow, ID, United States

**Keywords:** ε-polylysine (ε-PL), wood durability, bio-based wood preservative, leaching, mass loss, antifungal resistance

## Abstract

There has been a growing interest in seeking natural and biobased preservatives to prevent the wood from deteriorating during its service life, thereby prolonging carbon storage in buildings. This study aims to assess the *in vitro* and *in vivo* antifungal properties of epsilon poly-L-lysine (EPL), a secondary metabolite from *Actinomyces*, against four common wood-inhabiting fungi, including two brown-rot fungi, *Gloeophyllum trabeum* (*GT*) and *Rhodonia placenta* (*RP*), and two white-rot fungi, *Trametes versicolor* (*TV*) and *Irpex lacteus* (*IL*), which has rarely been reported. Our results indicate that these fungi responded differently due to EPL treatment. From the *in vitro* study, the minimal inhibitory concentration of EPL against *GT, TV*, and *IL* was determined to be 3 mg/ml, while that of *RP* was 5 mg/ml. EPL treatment also affects the morphology of fungal hyphae, changing from a smooth surface with a tubular structure to twisted and deformed shapes. Upon EPL treatment with wood samples (*in vivo*), it was found that EPL could possibly form hydrogen bonds with the hydroxy groups in wood and was uniformly distributed across the transverse section of the wood samples, as indicated by Fourier transform infrared spectroscopy and fluorescence microscopy analyses, respectively. Compared with control wood samples with a mass loss of over 15% across different fungi, wood samples treated with 1% EPL showed negligible or very low (<8%) mass loss. In addition, the thermal stability of EPL-treated wood was also improved by 50%. This study suggests that EPL could be a promising alternative to traditional metallic-based wood preservatives.

## Introduction

Wood-inhibiting fungi are important drivers in the carbon cycle of the earth by decomposing various organic matters into carbon dioxide and water (Treseder et al., 2016; Li et al., 2019), but they are also major decayers that have caused significant economic losses by deteriorating living trees and wood products (Oliva et al., 2014; Riquelme et al., 2018). For example, it has been estimated that 10% of the lumber cut each year is used to replace wooden building material that has decayed or molded (Morrell, 2018). Brown-rot and white-rot fungi are two of the most common decayers attacking different components in the wood. While brown-rot fungi feed on cellulose and hemicellulose, leaving the modified lignin behind, white-rot fungi selectively or simultaneously attack lignin and holocellulose (Goodell, 2003). These fungi can cause significant mechanical strength loss in wood at the incipient decay stage. For example, 50% toughness loss was observed when wood samples lost 1% weight loss (Richards, 1954), and 10% reduction in compressive strength when wood samples lost 2% of weight (Mizumoto, 1966). Approaches to protecting wood from various fungal damage include non-fungicidal (Guo et al., 2018), fungicidal (Cai et al., 2020), and biological methods (Yildiz et al., 2017). In particular, chemicals (i.e., copper-based wood preservatives) used in fungicidal formulations have been challenged by the ever increasing antimicrobial resistance of the fungi (Tang et al., 2016), strict environmental regulations (Adam et al., 2009), and the growing awareness of sustainable alternatives (Dong et al., 2020). These issues have led to the exploration of new antimicrobial agents to reduce or eliminate fungal deterioration of wood products.

Epsilon poly-L-lysine (EPL) is a secondary metabolite biosynthesized by *Streptomyces albulus* through industrial fermentation (Bankar and Singhal, 2013). It is a linear homopolymer consisting of 25–35 L-lysine residues, one of the essential amino acids. These L-lysine compounds are linked by peptide bonds (ε-amino bond), which are formed by an α-carboxylic acid group of L-lysine and an ε-amino group of another L-lysine (Adam et al., 2009; Dong et al., 2020). As a naturally produced polycationic peptide, EPL has excellent antimicrobial properties against a broad spectrum of microorganisms, including bacteria, yeast, molds, and fungi, under a wide pH range of 2–9 (Hiraki, 1999). For example, EPL was reported to inhibit the growth of gray mold caused by *Botrytis cinerea*, a common postharvest pathogenic fungus (Jiao et al., 2020). In another study, less disease severity was caused by *Xanthomonas euvesicatoria*, a Gram-negative phylum Proteobacteria, when tomato plants were sprayed with EPL solution (Rodrigues et al., 2020). Moreover, the biocidal activities of EPL are not affected by high temperature and can last for 20 min at 120°C. EPL is also readily soluble in water, editable, and biodegradable (Yoshida and Nagasawa, 2003). It is generally recognized as safe (GRAS Notice No. GRN 000135) by the Food and Drug Administration (FDA) in 2003. Based on its renewability, strong antimicrobial properties across a wide pH range, high-temperature resistance, and low toxicity, EPL has been extensively used in food preservation and biomedical industries (Hyde et al., 2019). However, the antifungal activity of EPL against wood-rotting fungi has not been reported.

The objective of this study was to examine the feasibility of using EPL as a potential biobased wood preservative for wood protection through *in vitro* and *in vivo* studies. First, the antifungal activities of EPL against four wood-decaying fungi, including two brown-rot fungi, *Gloeophyllum trabeum* (*GT*) and *Rhodonia placenta* (*RP*), and two white-rot fungi, *Trametes versicolor* (*TV*) and *Irpex lacteus* (*IL*), were identified using malt agar as substrate (*in vitro*). The effect of EPL on the fungal growth rate was monitored over 2 weeks, and the morphological changes were also presented. Then, the fungal resistance of EPL-treated wood samples was evaluated. Specifically, southern pine (*Pinus taeda* L.) and yellow poplar (*Liriodendron tulipifera L*.) sapwood samples were impregnated with different concentrations of EPL (1, 10, and 15%), and the distribution of EPL in wood was characterized. The interactions between EPL and wood were also determined using Fourier transform infrared spectroscopy and thermogravimetric analysis. A rigorous leaching test was conducted on the treated samples and these samples were exposed to three of the tested wood-decaying fungi (*RP, TV*, and *IL*). The mass loss due to leaching and fungal decay was reported.

## Materials and methods

### Materials

Epsilon poly-L-lysine (EPL, >95%, molecular weight is between 3,600 and 4,300) was purchased from MarkNature (Qingdao, China). Agar, yeast extract powder, and malt extract were obtained from Fisher Chemical. 4′,6-Diamidino-2-phenylindole dihydrochloride (DAPI) and Calcofluor White M2R (1 g/L) premixed with Evans blue (0.5 g/L) were purchased from MilliporeSigma. A total of two brown-rot fungi, *Gloeophyllum trabeum* (Madison 617, ATCC 11539) (*GT*) and *Rhodonia placenta* (Fr.) Niemelä et al. (ATCC#11538) (*RP*), and two white-rot fungi, *Trametes versicolor* (Linnaeus: Fries) Lloyd (ATCC#42462) (*TV*) and *Irpex lacteus (Fr.) Fr*. (ATCC#11245) (*IL*), were used throughout the study. Clear sapwood sample of southern pine (*Pinus taeda* L.) and yellow poplar (*Liriodendron tulipifera L*.) were used for brown-rot fungus and white-rot fungus decay tests, respectively. The sapwood was cut into 14 × 14 × 14 mm sections for wood treatment and durability test, and 3 × 28 × 34 mm as fungal feeder strips. The density of the wood durability testing samples after drying in a forced draft oven at 60°C was 473.89 kg/m^3^ (SD 38.25, *n* = 48) and 490.58 kg/m^3^ (SD 16.81, *n* = 96) for southern pine and yellow poplar samples, respectively. These samples were selected per the AWPA E10 standard (AWPA, 2016a).

### *In vitro* antifungal properties of epsilon poly-L-lysine

The antifungal activity of EPL against the wood-decaying fungi was assessed using malt agar as substrate. In brief, the malt agar solution was formulated with malt, agar, and yeast extract at a concentration of 2, 1.5, and 0.2%, respectively, using DI water as a solvent, followed by autoclaving at 120°C for 20 min. Then, part of the autoclaved malt agar solution was fully mixed with the EPL powder to obtain EPL-amended malt agar solutions at four different concentrations of 1, 2, 3, and 4 mg/ml in a biosafety hood. These concentrations were selected based on our preliminary study (unpublished) that evaluated appropriate EPL concentrations to effectively reduce fungal activity. Both the unamended and EPL-amended solutions were further transferred to Petri dishes (diameter of 90 mm) using sterilized syringes, with 12.5 ml cast per plate. After the malt agar solution solidified, an active growing fungus plug (*GT, RP, TV*, and *IL*) with an area of 100 mm^2^ was placed in the middle of each Petri dish. Then, the Petri dishes were sealed with parafilm and kept in a controlled chamber in the dark at a temperature of 25°C and relative humidity of 75%.

Fungal growth in the malt agar substrate was monitored daily for 14 days as it takes different fungi about 7–14 days to fully cover the whole Petri dish, depending on the fungal strains (Barbero-López et al., 2020). Photos of each Petri dish due to different EPL treatments, fungal species, and replicates were taken every day using a customized box to minimize the stray light in the photos and to obtain clear pictures (Supplementary Figure S1). The area covered by a fungus was measured using ImageJ software (National Institutes of Health, Bethesda, Maryland). All the treatments were carried out in five replicates. A total of 100 Petri dishes were prepared [5 replicates per treatment × 5 treatments (4 EPL treatments and one control) ×4 fungi (*GT, RP, TV*, and *IL*)]. The daily fungal growth rate was calculated using the following equation:


$$\begin{array}{l} \text{Daily fungal growth rate }\left(\text{\%}\right)=\;\frac{A_{day\#}-A_{plug}}{A_{\max}}\times 100\% \end{array}$$

where *A*_*day#*_ represents the area of the fungus covered on the Petri dish each day, *A*_*plug*_ means the area of the plug used to inoculate the plate, and *A*_*max*_ denotes the theoretical maximum area that could be covered by the fungus. In this study, the diameter of the plate is 90 mm, and the maximum area is 6,358.5 mm^2^.

### Microscopic observation of fungi growth in EPL-amended malt agar substrates

**Figure 1:**
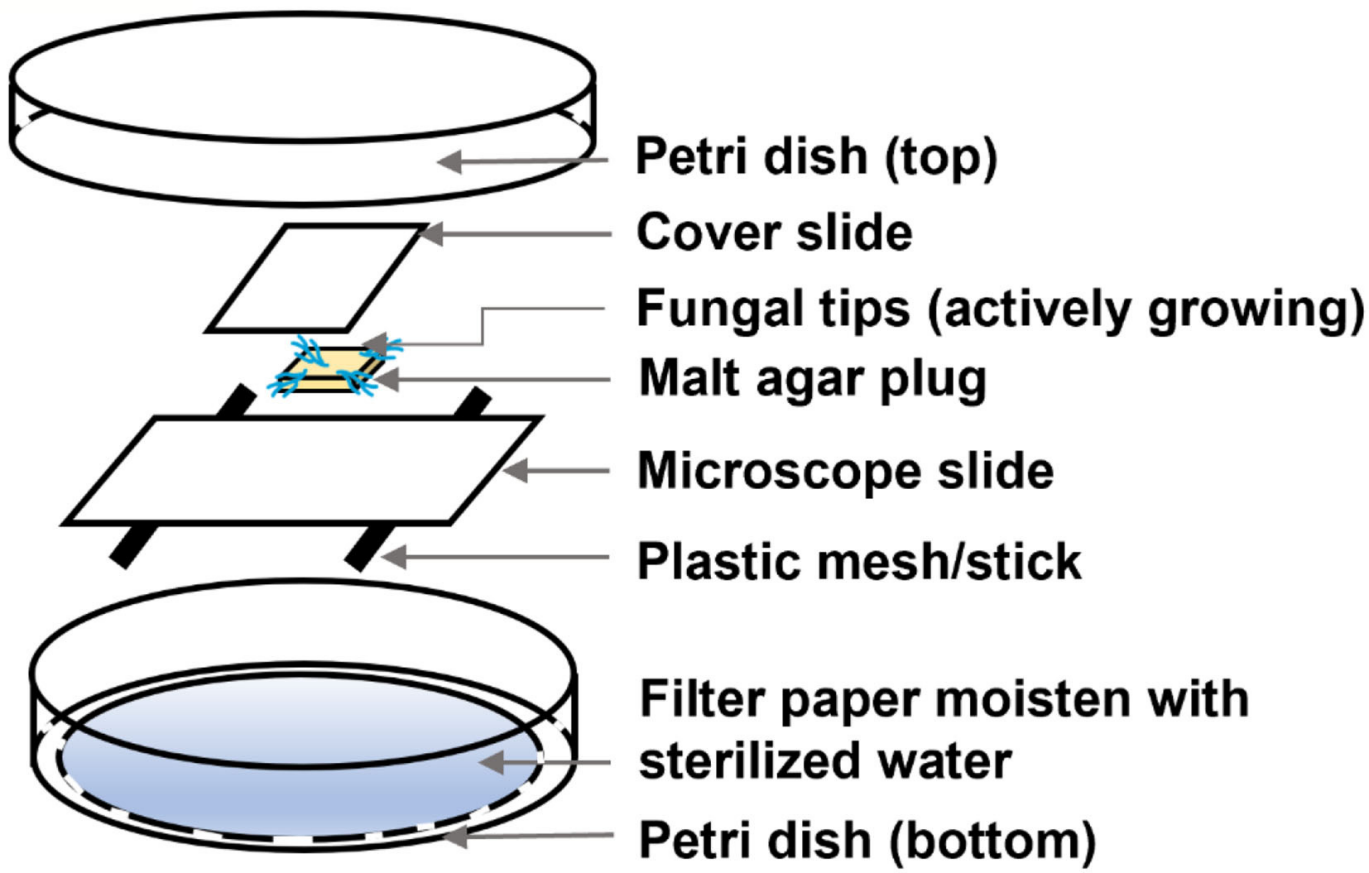
Expanded view of the fungal slide culture for microscopic observation.

The morphological changes due to the effect of EPL were observed using an Olympus microscope (BX51, Tokyo, Japan) under the magnification of 600X. The fungal slide culture was prepared as follows (Figure 1). The microscope slides, cover slides, and filter papers were autoclaved at 120°C for 20 min before further use. In brief, a filter paper was placed in a Petri dish (bottom) and wetted with 2 ml of sterilized DI water. Then, two spacers were placed between the filter paper and a microscope slide to create airflow, followed by placing a 5-mm^2^ solidified EPL-amended malt agar square in the middle of the slide. The EPL-amended malt agar substrate consists of 1 mg/ml for *GT*, 3 mg/ml for *RP*, and 2 mg/ml for both *TV* and *IL*, while the control malt agar substrate was prepared without adding EPL, as previously stated. Different EPL concentration levels were chosen for different fungal species based on their specific response to the external environment that could affect the fungal growth to a certain degree while still allowing for morphological change observation. The four corners of the malt agar plug were then inoculated with the active growing fungal tips (without adhering to the control malt agar substrate) and were further attached to a covering slide. The complete Petri dish set covered by a top plate was then sealed with parafilm and incubated at a temperature of 25°C and relative humidity of 75% in an environmental chamber in the dark for 2 days. The cover slides attaching with the newly grown fungal hyphae were then laid on another plain microscope slide and were fixed either by a drop of DI water or staining solutions for light microscopic or fluorescence microscopic (under blue light excitation) observation, respectively. For fungal culture staining, the samples were first exposed to a drop of DAPI (10 μg/ml), a cell-permeable dye that binds to the nucleus (Yun and Lee, 2016), for 10 min, followed by a drop of Calcofluor white and a drop of potassium hydroxide (10%) for another 10 min. Calcofluor white can attach to chitin, one of the major components of the fungal cell wall, while potassium hydroxide was added for better visualization of fungal elements (Denny et al., 2020). Excess solutions were removed using Kimber paper.

### Characterization of epsilon poly-L-lysine and EPL-treated wood

The sample powders, including EPL, untreated wood, and EPL-treated wood (15% EPL treatment on southern pine sapwood samples) samples, were freeze-dried using a freeze drier (Labconco, USA) for Fourier transform infrared (FTIR) spectroscopy analysis and thermogravimetric analysis (TGA), as stated in the following text. Specifically, the chemical changes of EPL treatment on wood were measured using FTIR spectroscopy (Nicolet IS 10, Thermo Scientific). The samples were collected in the wavenumber range of 400–4,000 *cm*^−1^ at a resolution of 4 *cm*^−1^. The obtained spectra were baseline corrected and normalized by using PerkinElmer software. The thermal stability of EPL-treated wood was studied using the thermogravimetric analysis (TGA 7, Perkin Elmer) technique under nitrogen. Approximately 5 mg of each sample was weighted on a platinum crucible and was heated from room temperature to 800°C at a heating rate of 10°C/min (Lin et al., 2018). Each sample was analyzed in duplicate, and the average data were presented.

### Preservative treatment by impregnation of wood in epsilon poly-L-lysine solutions

The wood samples were immersed in the treating solutions and were subjected to vacuum (1,000 mbar) for 20 min, followed by atmospheric pressure for 30 min. This procedure was repeated three times to ensure the full impregnation of EPL in wood. The treating solutions, which consisted of 0, 1, 10, and 15% EPL in deionized water, were formulated to examine how various amounts of EPL in wood affect the durability against fungi. All the treatments are in six replicates. A total of 144 wood samples were included for each wood species [6 replicates × 4 EPL concentrations (3 treatments and one control) × 3 fungi (*RP, TV*, and *IL*) × 2 (with leaching and without leaching]. The fungus *GT* was not included in this part of the study because of contamination during the durability test. These samples were dried at 60°C in a forced draft oven and were weighed to a precision of 0.01 g before the preservative treatment.

### Quantification of epsilon poly-L-lysine in wood by retention and mass gain

Retention and mass loss are two main indicators used to quantify the amount of chemicals in wood after preservative treatment. Specifically, retention is one of the most convenient indicators to estimate the amount of preservatives in wood by calculating the differences between the mass of the untreated samples under room conditions and the wet mass after treatment (Svedberg and Lindström, 2012), which is widely used in the wood preservation industry. In comparison, mass gain can accurately quantify the amount of preservative retained in wood after preservative treatment. It is obtained by calculating the oven-dried mass differences before and after treatment. This process is more tedious and could lead to the mitigation of chemicals from the inner part of the samples to the outer layer of samples (Dubey et al., 2012). Nevertheless, after the impregnating process, the surplus solution from the surface of the wood cubes was wiped, and the wet mass of the cube was immediately recorded. The cubes were further dried at 60°C for 48 h, and the corresponding mass was recorded. The retention and mass gain due to EPL treatments were calculated according to the following equations:


$$\begin{array}{l} \text{Retention }\left(\text{kg}/{\text{m}}^{3}\right)=GC/V\;\times \;10 \end{array}$$

where *G* represents the mass difference between the oven-dried samples before treatment and wet samples after impregnation (g), *C* means grams of EPL in 100 g of treating solution, and *V* is the volume of wood cubes in cm^3^.


$$\begin{array}{l} \text{Mass Gain }\left(\text{\%}\right)=\left(m_{A\text{-}trt}-m_{B\text{-}trt}\right)/m_{B\text{-}trt}\;\times \;100\% \end{array}$$

where *m*_*B*-*trt*_ and *m*_*A*-*trt*_ are the mass of wood samples dried at 60°C in a forced draft oven before and after treatment, respectively.

### Fluorescence microscopic analysis of treated wood

The micro-slides of both untreated and treated wood samples (~30 μ*m* in thickness) in cross-sections were cut by a microtome (AO Spencer No. 860) (Patel et al., 2013). The angle of the blade (knife) was adjusted to 30° to avoid distortion of the wood structure during the cutting process (Richards, 1942). The obtained cuts were mounted on the microscope slide with deionized water and were examined using a fluorescence microscope (BX 51/52 Olympus) under blue (BA 420 nm), red (BA 515 nm), and green (BA 590 nm) light, which was further composited into one image by using ImageJ software (Bankhead, 2014).

### Leaching of impregnated wood samples

Before the wood durability test, the samples were subjected to a leaching test according to the AWPA E11 (2016) standard (AWPA, 2016b). Specifically, wood samples were immersed in deionized (DI) water at a wood-to-water volume ratio of 1:2.5 and were shaken 100 rpm under room condition. The leachates were replaced with fresh deionized water at time intervals of 1, 6, 24, 48 h, and thereafter every 48 h until a total leachate collection of nine was obtained. After the leaching test, the wood cubes were removed from the beaker, and excess water on their surface was wiped off.

### Fungal resistance of epsilon poly-L-lysine-treated wood

Durability of EPL-treated wood against a brown-rot fungus (*RP*) and two white-rot fungi (*TV* and *IL*) was evaluated per AWPA E10 (2016) standard with some modifications (AWPA, 2016a): (1) malt agar medium in DI water containing malt, agar, and yeast extracts at concentrations of 2, 1.5, and 0.2%, respectively, was used as a substrate for the durability test, other than soil; (2) all wood cubes with and without leaching tests were sprayed with 70% of alcohol and dried in a biosafety hood for 30 min. This sterilization process was repeated three times before inoculation of the wood samples into the actively growing culture bottles. In short, wood feeder strips were pre-inoculated with either brown rot or white rot in the malt agar substrate. Then, the feeder strips with active fungal growth were transferred to another culture bottle with the malt agar substrate. The sterilized wood blocks were inoculated on the top of the feeder strips with cross-sections facing down. The culture bottles were sealed with parafilm and maintained in a conditioning chamber at a temperature of 25°C and a relative humidity of 75% for 8 weeks.

After 8-week incubation, the samples were removed from the culture bottles, and the fungal mycelia on the surface of the wood cubes were carefully brushed off to obtain dry mass at 60°C in a forced draft oven. The mass loss of wood samples due to decay were calculated based on the following equation:


$$\begin{array}{l} \text{Mass Loss }\left(\text{\%}\right)=\left(m_{B\text{-}f}-m_{A\text{-}f}\right)/m_{B\text{-}f}\;\times \;100\% \end{array}$$

where *m*_*B*-*f*_ and *m*_*A*-*f*_ are the oven-dried mass of a wood cube before and after being exposed to a wood-decaying fungus, respectively.

### Statistical analysis

The data of retention, mass gain after EPL treatment, and mass loss due to decay were subjected to statistical analysis using SAS software (version 9.4; SAS Institute, North Carolina, USA). These analyses include the normality test, homogeneity of variance test, and appropriate analysis of variance (ANOVA) method. For all our data, the homogeneity of variance is unequal, so a nonparametric *post-hoc* analysis approach (Games–Howell test) was used to compare all of the group differences. The confidence level was set at 95%.

## Results and discussion

### Wood-decaying fungal growth on EPL-amended malt agar substrates

**Figure 2:**
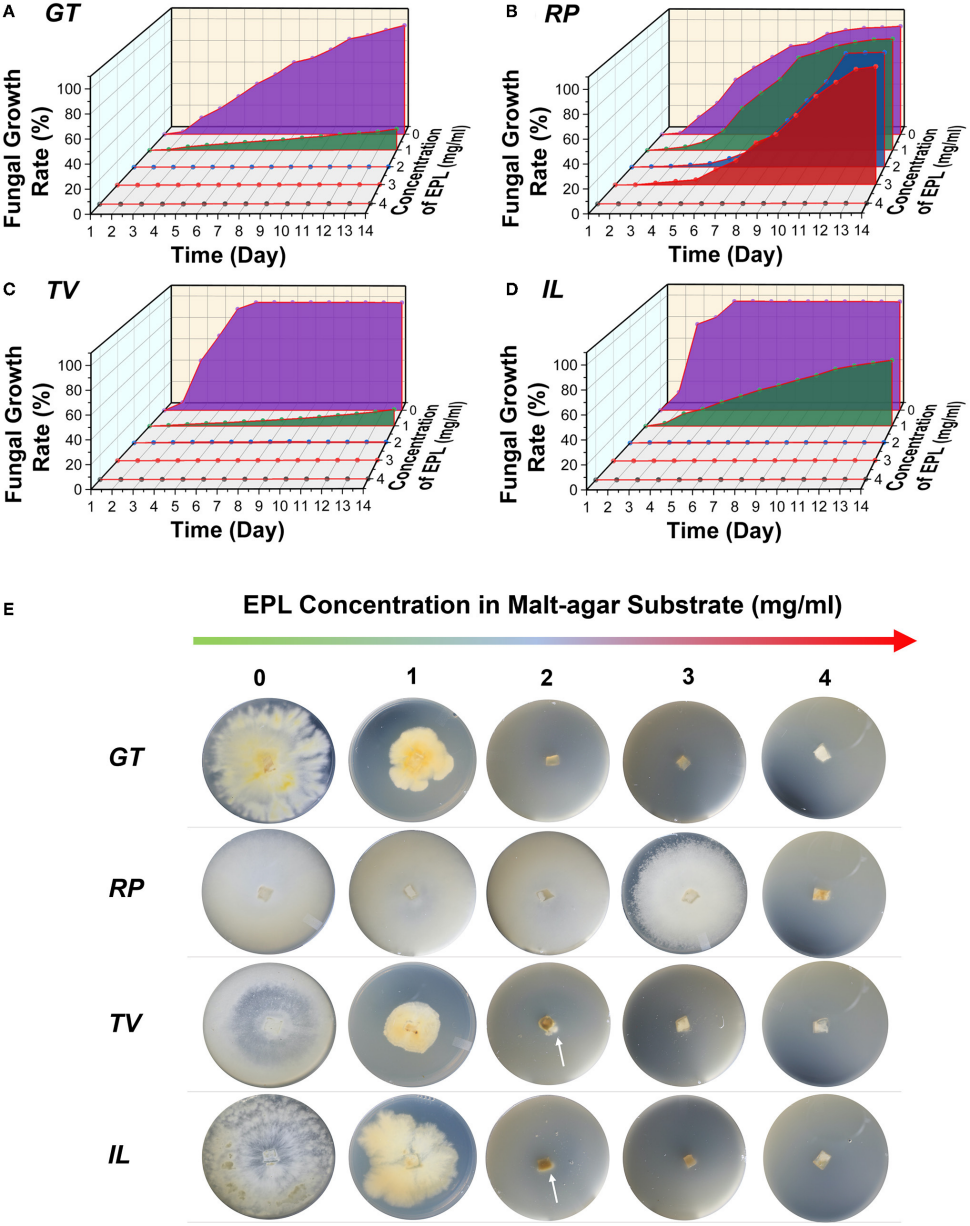
Average fungal growth rates of two brown-rot fungi, **(A)** *GT* and **(B)** *RP*, and two white-rot fungi, **(C)** *TV* and **(D)** *IL*, against different concentrations of EPL in malt agar substrates over a 14-day incubation period; **(E)** photographs of four wood-decaying fungi grew on malt agar substrates amended with 0, 1, 2, 3, and 4 mg/ml of EPL on day 14. The arrows in the Petri dishes represent mycelia colonization.

The effects of different concentrations of EPL on the average fungal growth rates of the two brown-rot fungi, *GT* and *RP*, and two white-rot fungi, *TV* and *IL*, in the malt agar substrate over a 14-day incubation period are shown in Figure 2. For control, it took *GT* and *RP* about 14 and 12 days, respectively, to fully cover the malt agar substrates. In comparison, the mycelia of the two white-rot fungi, *TV* and *IL*, completely colonized the control substrates on days 6 and 5, respectively, indicating these two white-rot fungi were more aggressive growers than the brown-rot fungi based on current laboratory conditions.

After EPL treatment, the four fungal strains responded differently to EPL-amended malt agar substrates, with *TV* being the most affected, while *RP* was the least sensitive to EPL. For example, at an EPL concentration of 1 mg/ml, the average fungal growth rates for *GT, RP, TV*, and *IL* were 19, 100, 15, and 59%, respectively, on day 14. The mycelial color also changed with EPL concentrations and became darker in *GT, TV*, and *IL* at 1 mg/ml (Figure 2E).

As the concentration of EPL increases to 2 mg/ml, the growth of *GT* was completely suppressed, while the growth of both *TV* and *IL* had initial growth at an early stage and then toward regression at a growth rate of < 0.5% on day 14 (Supplementary Figure S2). For example, *TV* and *IL* stopped growing on days 9 (Supplementary Figure S2) and 4 (Supplementary Figure S2), respectively, and at this stage, the hyphal tips of both fungi appeared normal. However, on day 14, condensation of the protoplasm was observed, which could be related to retroversion of the growth (Riquelme et al., 2018). By contrast, *RP* was very aggressive at EPL of 2 mg/ml, and its growth was not completely contained until EPL concentration reached 4 mg/ml. Although EPL of 2 mg/ml completely or almost retarded the growth of *GT, TV*, and *IL*, some replicates from these fungi continued growing after a 2-month checkup of the Petri dishes (i.e., for *GT, two* of five replicates continue growing; for *TV*, all five of the replicates continue growing, and for *IL, four* of five grew). A similar phenomenon was also observed in *RP* (two of five replicates continued growing) under EPL of 4 mg/ml. It is worth noting that no obvious hyphal expansion was seen in *GT, TV*, and *IL* at 3 mg/ml and *RP* at 5 mg/ml after a 2-month follow-up examination. Together with our preliminary study (unpublished), the minimal inhibitory concentration of EPL against all the fungi was determined to be 3 mg/ml, except for *RP*, which is 5 mg/ml. Similar research has reported that *RP* is least sensitive to different organic acids (Barbero-López et al., 2020). Our results are also consistent with previous research that EPL treatment causes a dose–response effect on various fungi, such as necrotrophic pathogenic fungi, *Sclerotinia sclerotiorum* and *Botrytis cinerea*, and fruit pathogens, *Penicillium expansum* and *Colletotrichum gloeosporioides* (Zhou et al., 2021; Bai et al., 2022; Lv et al., 2022).

### Microscopic observations of fungi morphology due to the effects of EPL

**Figure 3:**
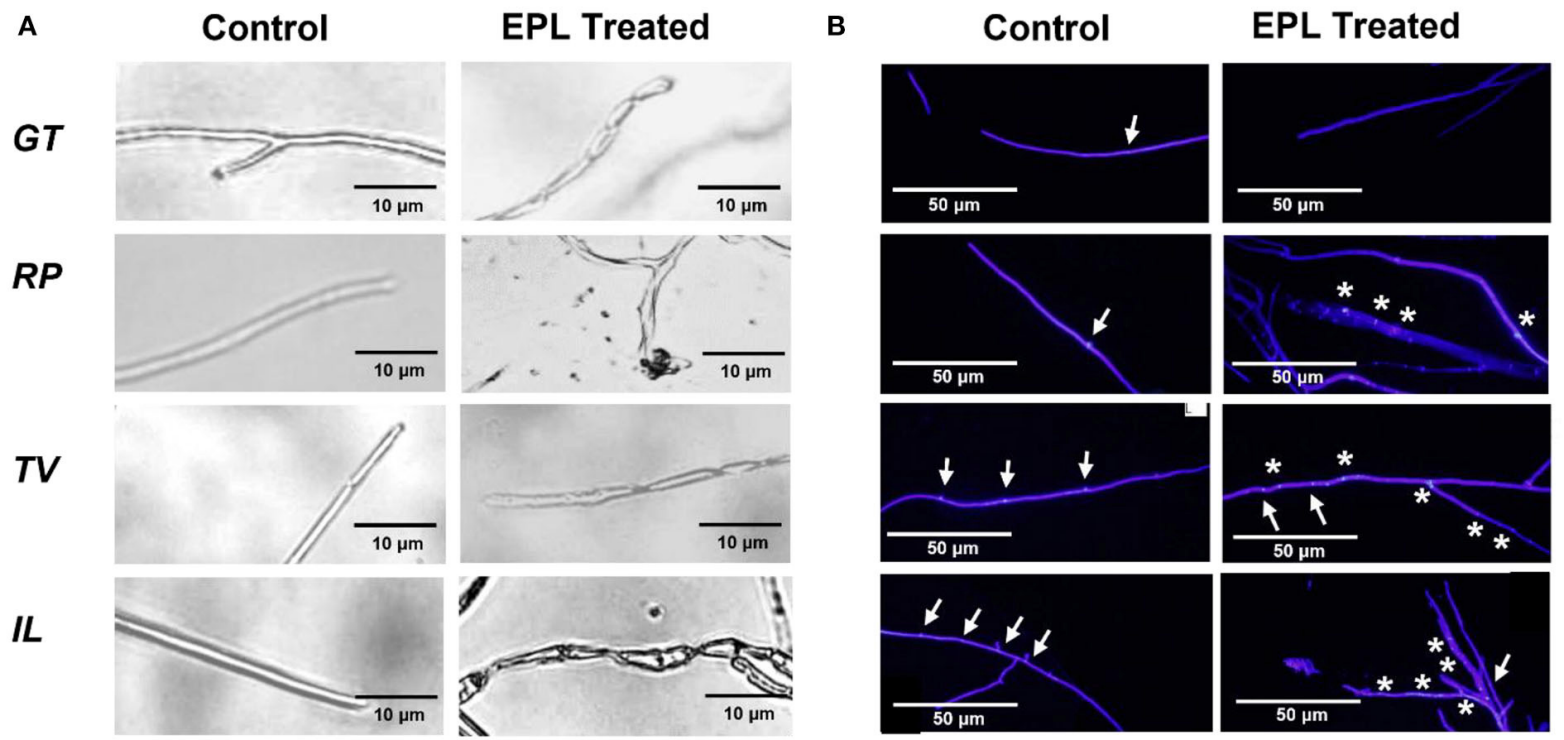
**(A)** Optical micrographs showing morphological changes of the hyphae before and after the EPL treatment and **(B)** fluorescent micrographs of four fungi, *GT, RP, TV*, and *IL*; intact nuclei are indicated by arrowheads (↑), while fragmented nuclei are indicated by *. The actively growing fungal tips were exposed to the control malt agar substrate or amended with 1 mg/ml EPL for GT, 3 mg/ml EPL for RP, and 2 mg/ml EPL for both TV and IL.

The morphological changes caused by EPL were obtained by microscopic observations, as shown in Figures 3A,B. Significant differences in hyphae growth between the control and EP-amended malt agar were observed on all the fungal strains. In the control group, the hyphae displayed a smooth surface with a tubular structure. By contrast, the hyphae of all the fungi that grew on EPL-amended malt agar substrates showed twisted or distorted shapes. The exposure of *RP* to EPL also triggers cell leakages. Similar research has been reported that when a fungus is exposed to a fungicide, it will induce apoptotic-like death, such as shrinkage, leakage, and rough surface (Chaves-Lopez et al., 2018; Hou et al., 2020).

Figure 3B shows the fluorescence micrographs of the fungi exposed to control and EPL-amended malt agar substrate on day 9. In the control group, intact nuclei were detected for the four fungi. In comparison, different fungi responded differently to EPL treatment and led to various changes in the nucleus. For example, in *GT*, the nuclei or debris of nuclei were not observed and were likely dissolved in the cytoplasm, indicating the high vulnerability of this species (Morris, 1975). This observation is also consistent with the low growth rate of *GT* at an EPL concentration of 1 mg/ml. In comparison, part of the nuclei fragmented in the remaining three fungal stains upon EPL treatment (Gao et al., 2019), and some of them remain unchanged. These results could explain why *RP* and *IL* were less sensitive to EPL treatment at 1 mg/ml. Although TV was very sensitive to EPL concentrations at 1 mg/ml or higher during the two-week growth observation, its regrowth after a 2-month check was possibly related to the undamaged nucleus that remained in the hyphal tips.

### Interaction between epsilon poly-L-lysine and wood and their thermostability

**Figure 4:**
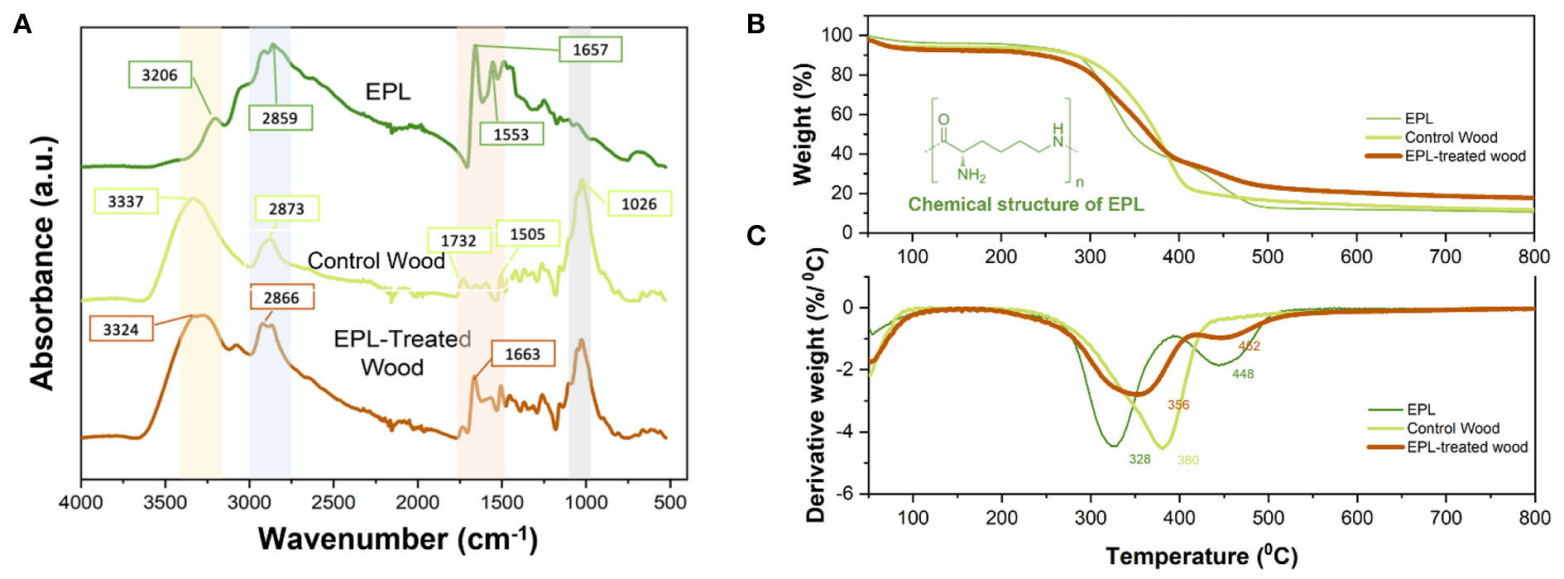
**(A)** Infrared spectra, **(B)** thermal gravimetric (TG) analysis, and **(C)** derivative TG of EPL, control, and EPL-treated wood samples.

The ATR-FTIR spectra of EPL, control wood (southern pine), and EPL-treated wood samples are shown in Figure 4A. The FTIR spectrum of EPL exhibits a wide absorption band between 2,500 and 3,590 cm^−1^ due to the abundant NH, NH_2_, CH, and CH_2_ groups in its molecular chains (Zhu et al., 2010). Specifically, the peaks at 3,420 and 3,206 cm^−1^ are attributed to the stretching of primary amine (-NH_2_) and secondary amine (-CONH-), respectively. The peaks at 2,930 and 2,859 cm^−1^ are attributed to the CH stretching of the CH_2_ and CH groups, respectively. In addition, the characteristic absorptions at 1,657, 1,553, and 1,250 cm^−1^ are corresponding to the C=O stretching, NH bending (primary amine), and C-N stretching of amine, respectively. The N-H wagging of primary and secondary amines is also observed at 951 and 688 cm^−1^, respectively.

In terms of the IR spectrum of the control wood specimen, strong and wide bands centered at 3,337 and 2,873 cm^−1^ correspond to O-H stretching vibration and C-H stretching, respectively, from various wood components, such as cellulose, hemicellulose, and lignin. The bands at 1,732 and 1,505 cm^−1^ are related to C=O stretching of the carboxylic group in hemicellulose and C=C stretching vibration in the aromatic ring of lignin, respectively. The strong and sharp band at 1,026 cm^−1^ is attributed to C-O stretching and C-H deformation in the guaiacyl unit of polysaccharide and lignin (Pandey and Theagarajan, 1997).

After EPL treatment, several changes were observed in the IR spectrum of EPL-treated wood samples. Specifically, O-H stretching at 3,337 cm^−1^ and C-H stretching vibration at 2,873 cm^−1^ from wood shifted to 3,324 and 2,866 cm^−1^, respectively. These changes are possibly related to the interactions between the hydroxyl groups of wood and amino groups of EPL. In addition, the C=O band at 1,657 cm^−1^ from EPL was shifted to 1,663 cm^−1^ in EPL-treated wood due to the formation of hydrogen bonds (Gao et al., 2014).

The TGA and derivative thermogravimetry (DTG) curves of EPL, control, and EPL-treated wood samples are shown in Figure 4B. In the first stage (room temperature to 100°C), the weight loss of all three treatments was mainly related to the evaporation of moisture (Svedberg and Lindström, 2012; Lin et al., 2018). As temperature increases, the EPL sample undergoes a mass loss of <10% before 280°C and a mass loss of ~67% in the temperature range of 280–500°C, with the latter having two peaks centered at 328 and 444°C (Figure 4C) due to the decomposition of EPL chains. The control wood sample was also rapidly decomposed during this temperature range, and its DTG curve shows a shoulder peak and a sharp peak at 350 and 380°C due to the degradation of hemicellulose and cellulose, respectively (González-Díaz and Alonso-López, 2017). In comparison, EPL-treated wood has a higher mass loss rate than that of EPL and control wood before 300°C and exhibits the lowest mass loss after 400°C. Moreover, the major decomposition peak of control wood at 380°C shifted to a lower temperature of 352°C in the EPL-treated wood. The charring rate of EPL-treated wood is 53% higher than that of the untreated wood (17.3 vs. 11.3%). These results indicated that EPL could shift wood degradation to the low-temperature pathway, thus improving the thermal stability of wood.

### Epsilon poly-L-lysine in wood

**Figure 5:**
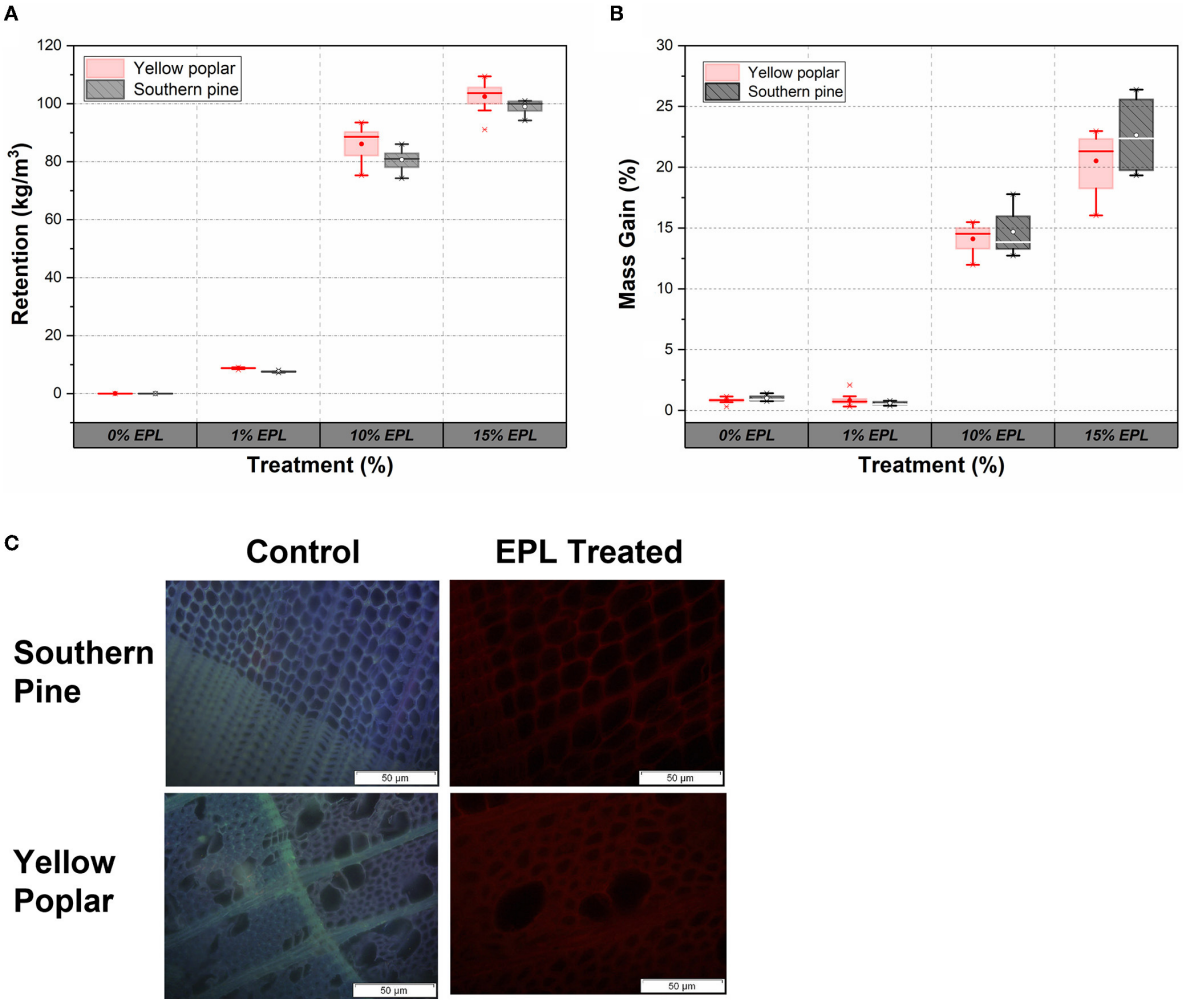
**(A)** Retention and **(B)** mass gain of hardwood (yellow poplar) and softwood (southern pine) under different concentrations of EPL treatment and **(C)** distribution of EPL in southern pine and poplar samples under fluorescence microscopy.

The retention and mass gain due to EPL treatment effects are presented in Figures 5A,B. Generally, retention and mass gain increased as a function of EPL concentration. For example, the retentions of EPL in yellow poplar samples at 1, 10, and 15% were around 9, 86, and 102 kg/m^3^, respectively, while those in southern pine samples at 1, 10, and 15% were 8, 81, and 99 kg/m^3^, respectively. Similar results were obtained in terms of mass gain, ranging from 1 to 25%. Also, based on the retention and mass gain results, there were no significant differences between southern pine and yellow poplar wood samples, except at an EPL concentration of 1% (*p* < 0.05).

The distribution of EPL along the cross-section of the wood samples under a fluorescence microscope is shown in Figure 5C. The untreated wood showed both blue and purple colors in the composited picture (Echard et al., 2015), whereas the EPL-treated wood was in red. The difference in colors could be related to the fact that different energy states of various chemical molecules in wood emit different colors under the fluorescence microscope (Richards, 1942; Patel et al., 2013), indicating the presence of EPL in wood.

### Decay resistance of epsilon poly-L-lysine-treated wood

**Figure 6:**
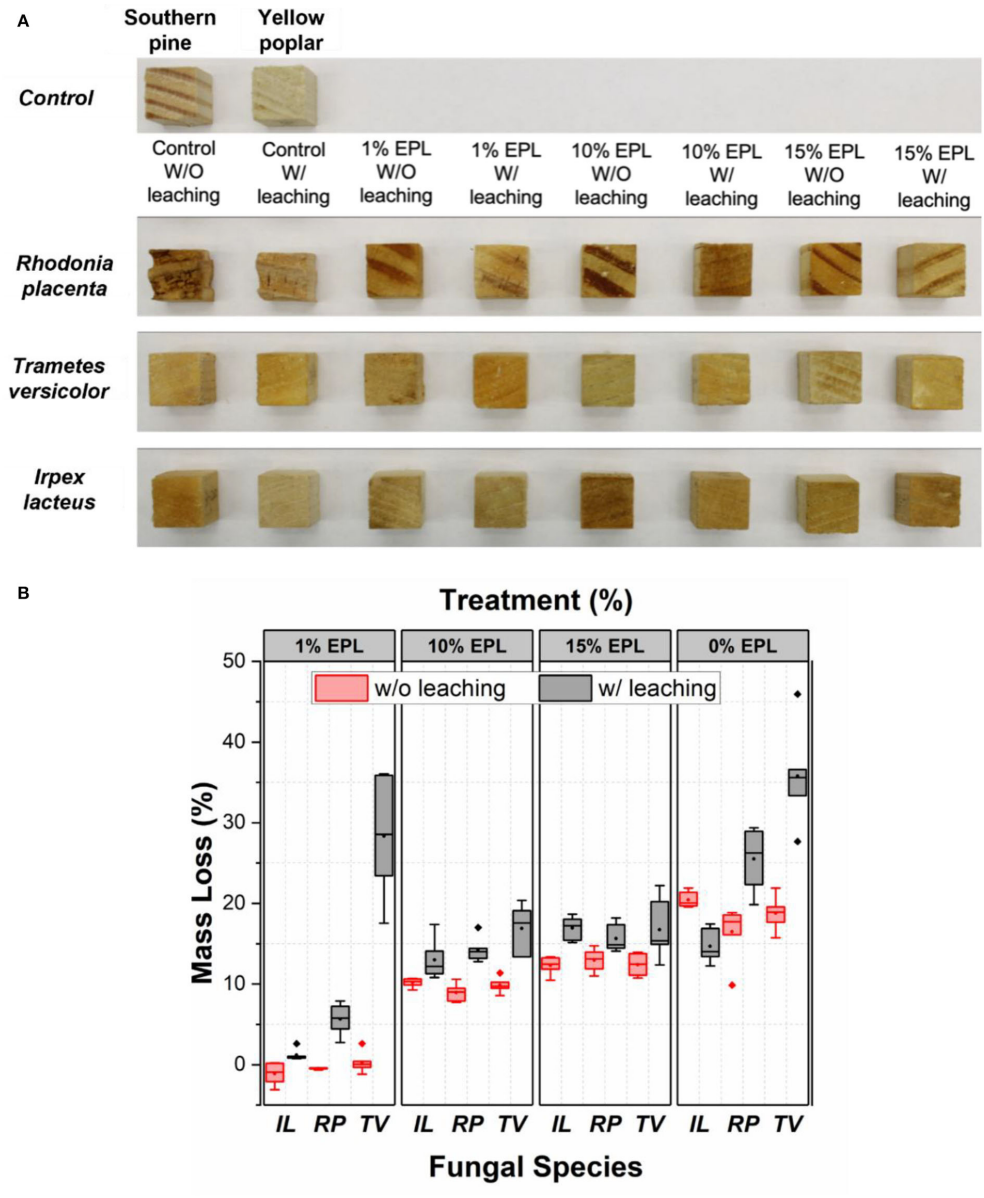
**(A)** Photos and **(B)** mass loss of wood cubes with different treatments after 8 weeks of fungal exposure. In total, six replicates were used. The brown-rot fungus *R. placenta* was inoculated into southern pine samples, while white-rot fungi, *T. versicolor* and *I. lacteus*, were inoculated on yellow poplar samples.

The photographic picture of the representative samples before and after the durability test are shown in Figure 6A. As compared with the samples either from the control group before fungal exposure or with EPL treatment after decay, wood cubes in the control groups due to *RP* decay showed extensive damage. However, the decay of control groups due to *TV* and *IL* cannot be easily visualized.

Figure 6B shows a box and whisker plot of mass loss of wood samples caused by fungal decay, which is significantly affected by EPL treatment. Especially, at an EPL concentration of 1% and regardless of the leaching test, the treated sample showed a significantly lower mass loss for all the fungi tested (*p* < *0.05*), except for *TV*, than for the water-treated wood cubes. This means even though part of EPL was removed during the leaching test, EPL remained in wood was still effective against *RP* and *IL*, but not against *TV*. The high mass loss of *TV*-exposed samples after leaching (28%) is possibly related to its partially undamaged nuclei, which allow *TV* to continue attacking wood (Kuo, 2021).

As the concentration of EPL increased to 10 and 15%, significantly higher mass losses were observed in the unleached samples than in the leached samples for all the tested fungal species (*p* < *0.05*). This observation is likely related to the leaching of EPL in the unleached samples (yellowish liquid) during the durability test, as shown in Supplementary Figure S3. On the contrary, the significantly lower mass losses (*p* < *0.05*) observed in the leached samples at high EPL indicate that the remaining EPL in wood after the leaching test can still provide adequate fungal resistance to these wood-decaying fungi. The negative mass loss here was unknown and could be possibly related to the residual hyphae mass in the wood after the durability test. Nevertheless, our 1% EPL treatment on wood (retention level of 7 kg/m^3^) is comparable with current research findings by using ACQ and tannin–boron formulations (Ung and Cooper, 2005; Wieland et al., 2012; Vasileva et al., 2017). By further refining the formulation, it could be possibly used for Use Category 4C: ground contact or freshwater (AWPA, 2017).

## Conclusion

This study demonstrated the effectiveness of using EPL as a potential alternative for wood protection. The minimal inhibitory concentration of EPL against GT, TV, and IL was found to be 3 mg/ml, while that of RP was 5 mg/ml. The morphology of the hyphae due to EPL treatment was changed from a smooth surface with a tubular structure to a twisted and deformed shape. When treating wood with EPL solutions, it was found that EPL can easily penetrate wood, resulting in a uniform distribution in wood. The thermal stability of EPL-treated wood was improved by 50%. EPL-treated wood generally showed improved decay resistance against common wood-decaying fungi at 1% level treatment, regardless of the rigorous leaching test. Future research should focus on treating larger wood samples and exposing them to more fungal species.

## Data availability statement

The raw data supporting the conclusions of this article will be made available by the authors, without undue reservation.

## Author contributions

Conceptualization, validation, resources, supervision, project administration, and funding acquisition: LC. Methodology, formal analysis, investigation, writing—original draft preparation, and writing—review and editing: LC and C-JK. Both authors have read and agreed to the published version of the manuscript.

## Funding

This project was supported by the USDA McIntire-Stennis Project (Accession Number 1021498).

## Conflict of interest

The authors declare that the research was conducted in the absence of any commercial or financial relationships that could be construed as a potential conflict of interest.

## Publisher's note

All claims expressed in this article are solely those of the authors and do not necessarily represent those of their affiliated organizations, or those of the publisher, the editors and the reviewers. Any product that may be evaluated in this article, or claim that may be made by its manufacturer, is not guaranteed or endorsed by the publisher.

## Author disclaimer

Any opinions, findings, conclusions, or recommendations expressed in the publication are those of the authors and do not necessarily reflect the view of the U.S. Department of Agriculture.
