# Supplementary material for: Epsilon poly-L-lysine as a novel antifungal agent for sustainable wood protection
Source: Front Microbiol. 2022 Sep 7;13:908541. doi: 10.3389/fmicb.2022.908541 (PMC9490314; doi:10.3389/fmicb.2022.908541)
Supplement: Supplementary file 1 [file Data_Sheet_1.PDF]

# Supporting Information

## Epsilon Poly L-lysine as a Novel Antifungal Agent for Sustainable Wood Protection

Lili Cai*, Chi-Jui Kuo

Department of Forest, Rangeland and Fire Sciences, University of Idaho, 875 Perimeter Drive, Moscow, ID 83844, USA

*Corresponding author: lcai@uidaho.edu

Number of pages: 14

Number of Figures: 3

Number of Tables: 5

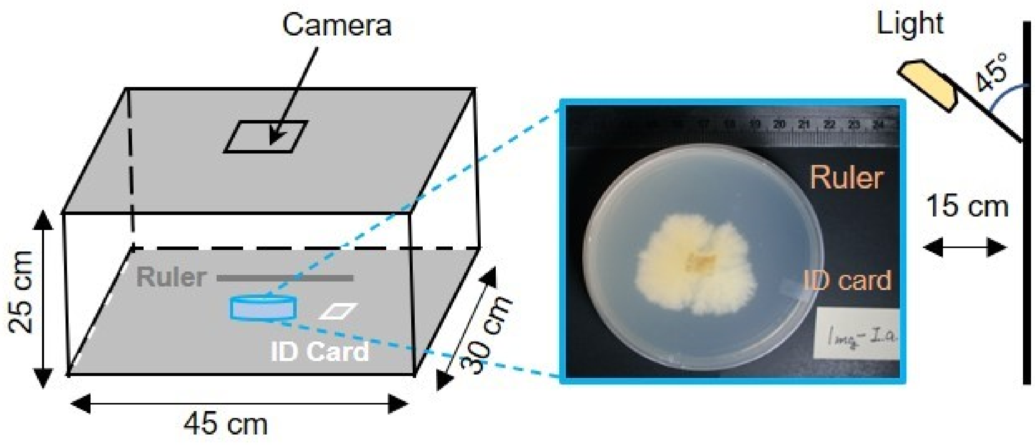


*Fig. S1 Schematic drawing of the customized box (left), the layout of a Petri dish, ruler and sample ID card inside the box (middle) and light source (right) for photo taking*

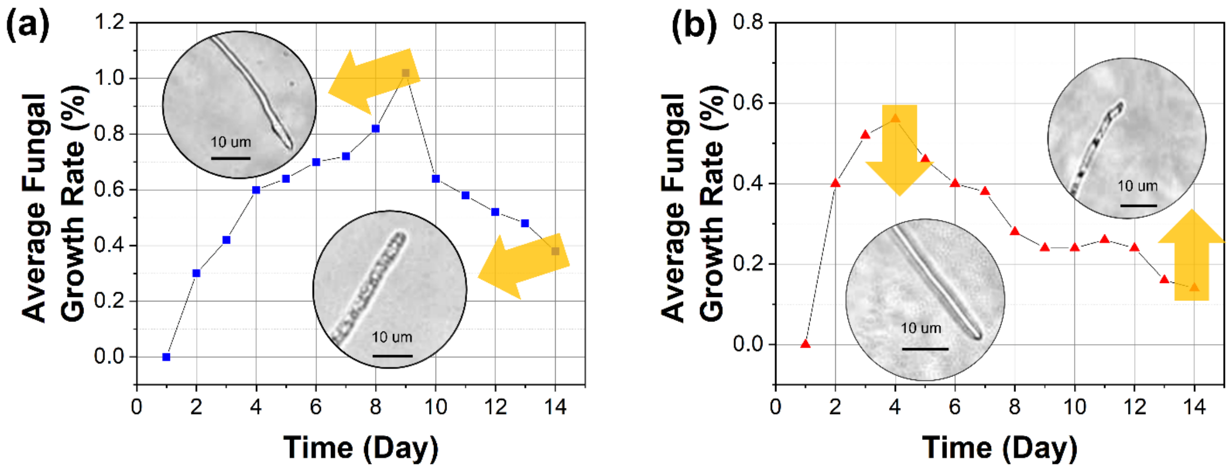


*Fig. S2. Growth of (a) TV and (b) IL at 2mg/ml of EPL amended malt agar substrate.*

# Supporting Information

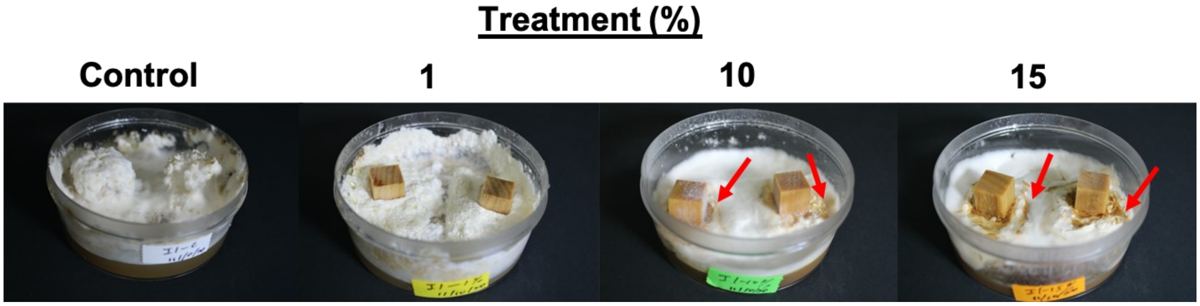


*Fig. S3 Culture bottles containing un-leached samples under wood durability test at different EPL treatments levels. The red arrow indicated that EPL solution leached out at the EPL treatment at 10% and 15%.*

*Table 1 SAS Code for Mass Gain and Retention Analysis*

| Code | Treatment | Wood Species |
|---|---|---|
| 1 | Control | Yellow poplar |
| 2 | Control | Southern yellow pine |
| 3 | 1% EPL | Yellow poplar |
| 4 | 1% EPL | Southern yellow pine |
| 5 | 10% EPL | Yellow poplar |
| 6 | 10% EPL | Southern yellow pine |
| 7 | 15% EPL | Yellow poplar |
| 8 | 15% EPL | Southern yellow pine |

# Supporting Information

*Table 2 Differences of Least Squares Means of Mass Gain (Adjusted P value < 0.05 means these two treatments are statistically different)*

| Differences of Least Squares Means | | | | | | | | | |
|---|---|---|---|---|---|---|---|---|---|
| Effect | treatment | _treatment | Estimate | Standard Error | DF | t Value | Pr > \|t\| | Adjustment | Adj P |
| treatment | 1 | 2 | -0.2000 | 0.08037 | 19.3 | -2.49 | 0.0221 | Tukey-Kramer | 0.2538 |
| treatment | 1 | 3 | -0.02083 | 0.08715 | 35.3 | -0.24 | 0.8124 | Tukey-Kramer | 1.0000 |
| treatment | 1 | 4 | 0.2333 | 0.06025 | 27.9 | 3.87 | 0.0006 | Tukey-Kramer | 0.0168 |
| treatment | 1 | 5 | -11.5500 | 0.1865 | 25.4 | -61.94 | <.0001 | Tukey-Kramer | <.0001 |
| treatment | 1 | 6 | -11.9917 | 0.4038 | 11.2 | -29.70 | <.0001 | Tukey-Kramer | <.0001 |
| treatment | 1 | 7 | -16.1917 | 0.3026 | 23.9 | -53.52 | <.0001 | Tukey-Kramer | <.0001 |
| treatment | 1 | 8 | -17.6000 | 0.5690 | 11.1 | -30.93 | <.0001 | Tukey-Kramer | <.0001 |
| treatment | 2 | 3 | 0.1792 | 0.1026 | 31.8 | 1.75 | 0.0905 | Tukey-Kramer | 0.6597 |
| treatment | 2 | 4 | 0.4333 | 0.08103 | 18.6 | 5.35 | <.0001 | Tukey-Kramer | 0.0006 |
| treatment | 2 | 5 | -11.3500 | 0.1942 | 28.8 | -58.45 | <.0001 | Tukey-Kramer | <.0001 |
| treatment | 2 | 6 | -11.7917 | 0.4074 | 11.6 | -28.94 | <.0001 | Tukey-Kramer | <.0001 |
| treatment | 2 | 7 | -15.9917 | 0.3074 | 25.3 | -52.03 | <.0001 | Tukey-Kramer | <.0001 |
| treatment | 2 | 8 | -17.4000 | 0.5715 | 11.3 | -30.44 | <.0001 | Tukey-Kramer | <.0001 |
| treatment | 3 | 4 | 0.2542 | 0.08776 | 33 | 2.90 | 0.0067 | Tukey-Kramer | 0.1247 |
| treatment | 3 | 5 | -11.5292 | 0.1971 | 30.9 | -58.50 | <.0001 | Tukey-Kramer | <.0001 |
| treatment | 3 | 6 | -11.9708 | 0.4088 | 11.8 | -29.28 | <.0001 | Tukey-Kramer | <.0001 |
| treatment | 3 | 7 | -16.1708 | 0.3092 | 26 | -52.30 | <.0001 | Tukey-Kramer | <.0001 |
| treatment | 3 | 8 | -17.5792 | 0.5725 | 11.4 | -30.70 | <.0001 | Tukey-Kramer | <.0001 |
| treatment | 4 | 5 | -11.7833 | 0.1868 | 25.5 | -63.10 | <.0001 | Tukey-Kramer | <.0001 |
| treatment | 4 | 6 | -12.2250 | 0.4039 | 11.3 | -30.27 | <.0001 | Tukey-Kramer | <.0001 |
| treatment | 4 | 7 | -16.4250 | 0.3027 | 23.9 | -54.26 | <.0001 | Tukey-Kramer | <.0001 |
| treatment | 4 | 8 | -17.8333 | 0.5691 | 11.1 | -31.34 | <.0001 | Tukey-Kramer | <.0001 |
| treatment | 5 | 6 | -0.4417 | 0.4408 | 15.6 | -1.00 | 0.3316 | Tukey-Kramer | 0.9689 |
| treatment | 5 | 7 | -4.6417 | 0.3504 | 37.9 | -13.25 | <.0001 | Tukey-Kramer | <.0001 |
| treatment | 5 | 8 | -6.0500 | 0.5958 | 13.3 | -10.15 | <.0001 | Tukey-Kramer | <.0001 |
| treatment | 6 | 7 | -4.2000 | 0.5011 | 23.2 | -8.38 | <.0001 | Tukey-Kramer | <.0001 |
| treatment | 6 | 8 | -5.6083 | 0.6952 | 19.8 | -8.07 | <.0001 | Tukey-Kramer | <.0001 |
| treatment | 7 | 8 | -1.4083 | 0.6417 | 17.3 | -2.19 | 0.0421 | Tukey-Kramer | 0.3937 |

# Supporting Information

*Table 3 Differences of Least Squares Means of Retention (Adjusted P value < 0.05 means these two treatments are statistically different)*

| Differences of Least Squares Means | | | | | | | | | |
|---|---|---|---|---|---|---|---|---|---|
| Effect | treatment | _treatment | Estimate | Standard Error | DF | t Value | Pr > \|t\| | Adjustment | Adj P |
| treatment | 3 | 4 | 1.2054 | 0.07993 | 20.3 | 15.08 | <.0001 | Tukey-Kramer | <.0001 |
| treatment | 3 | 5 | -77.3467 | 1.1747 | 23.1 | -65.85 | <.0001 | Tukey-Kramer | <.0001 |
| treatment | 3 | 6 | -71.9246 | 1.0263 | 11 | -70.08 | <.0001 | Tukey-Kramer | <.0001 |
| treatment | 3 | 7 | -93.6842 | 0.8202 | 23.1 | -114.22 | <.0001 | Tukey-Kramer | <.0001 |
| treatment | 3 | 8 | -90.2446 | 0.6798 | 11.1 | -132.76 | <.0001 | Tukey-Kramer | <.0001 |
| treatment | 4 | 5 | -78.5521 | 1.1758 | 23.2 | -66.81 | <.0001 | Tukey-Kramer | <.0001 |
| treatment | 4 | 6 | -73.1300 | 1.0276 | 11.1 | -71.17 | <.0001 | Tukey-Kramer | <.0001 |
| treatment | 4 | 7 | -94.8896 | 0.8219 | 23.3 | -115.46 | <.0001 | Tukey-Kramer | <.0001 |
| treatment | 4 | 8 | -91.4500 | 0.6817 | 11.2 | -134.14 | <.0001 | Tukey-Kramer | <.0001 |
| treatment | 5 | 6 | 5.4221 | 1.5586 | 32.2 | 3.48 | 0.0015 | Tukey-Kramer | 0.0179 |
| treatment | 5 | 7 | -16.3375 | 1.4314 | 41.1 | -11.41 | <.0001 | Tukey-Kramer | <.0001 |
| treatment | 5 | 8 | -12.8979 | 1.3558 | 33.2 | -9.51 | <.0001 | Tukey-Kramer | <.0001 |
| treatment | 6 | 7 | -21.7596 | 1.3124 | 24.7 | -16.58 | <.0001 | Tukey-Kramer | <.0001 |
| treatment | 6 | 8 | -18.3200 | 1.2295 | 19.1 | -14.90 | <.0001 | Tukey-Kramer | <.0001 |
| treatment | 7 | 8 | 3.4396 | 1.0636 | 33 | 3.23 | 0.0028 | Tukey-Kramer | 0.0322 |

*Table 4 SAS Code for Mass loss analysis*

| Fungus | Treatment | W/ or W/O leaching | SAS Code | Fungus | Treatment | W/ or W/O leaching | SAS Code |
|---|---|---|---|---|---|---|---|
| TV | Control | non-leaching | 1 | TV | 10% EPL | non-leaching | 13 |
| TV | Control | leaching | 2 | TV | 10% EPL | leaching | 14 |
| IL | Control | non-leaching | 3 | IL | 10% EPL | non-leaching | 15 |
| IL | Control | leaching | 4 | IL | 10% EPL | leaching | 16 |
| Pp | Control | non-leaching | 5 | Pp | 10% EPL | non-leaching | 17 |
| Pp | Control | leaching | 6 | Pp | 10% EPL | leaching | 18 |
| TV | 1% EPL | non-leaching | 7 | TV | 15% EPL | non-leaching | 19 |
| TV | 1% EPL | leaching | 8 | TV | 15% EPL | leaching | 20 |
| IL | 1% EPL | non-leaching | 9 | IL | 15% EPL | non-leaching | 21 |
| IL | 1% EPL | leaching | 10 | IL | 15% EPL | leaching | 22 |

| | | | | | | | |
|---|---|---|---|---|---|---|---|
| Pp | 1% EPL | non-leaching | 11 | Pp | 15% EPL | non-leaching | 23 |
| Pp | 1% EPL | leaching | 12 | Pp | 15% EPL | leaching | 24 |

*Table 5* Differences of Least Squares Means of Mass Loss (Adjusted P value < 0.05 means these two treatments are statistically different)

# Supporting Information

| Differences of Least Squares Means | | | | | | | | | |
|---|---|---|---|---|---|---|---|---|---|
| Effect | treatment | _treatment | Estimate | Standard Error | DF | t Value | Pr > \|t\| | Adjustment | Adj P |
| treatment | 1 | 2 | -17.1600 | 2.5139 | 6.26 | -6.83 | 0.0004 | Tukey-Kramer | 0.0081 |
| treatment | 1 | 3 | -1.6317 | 0.9317 | 7 | -1.75 | 0.1234 | Tukey-Kramer | 0.9477 |
| treatment | 1 | 4 | 4.1483 | 1.1609 | 9.96 | 3.57 | 0.0051 | Tukey-Kramer | 0.2459 |
| treatment | 1 | 5 | 2.2617 | 1.5850 | 8.45 | 1.43 | 0.1895 | Tukey-Kramer | 0.9904 |
| treatment | 1 | 6 | -6.4150 | 1.8791 | 7.4 | -3.41 | 0.0103 | Tukey-Kramer | 0.2906 |
| treatment | 1 | 7 | 18.5317 | 0.9897 | 8.21 | 18.72 | <.0001 | Tukey-Kramer | <.0001 |
| treatment | 1 | 8 | -9.3833 | 3.5479 | 5.6 | -2.64 | 0.0409 | Tukey-Kramer | 0.5951 |
| treatment | 1 | 9 | 19.8417 | 0.9851 | 8.13 | 20.14 | <.0001 | Tukey-Kramer | <.0001 |
| treatment | 1 | 10 | 17.7000 | 0.9418 | 7.23 | 18.79 | <.0001 | Tukey-Kramer | <.0001 |
| treatment | 1 | 11 | 19.0917 | 0.8487 | 5.03 | 22.50 | <.0001 | Tukey-Kramer | <.0001 |
| treatment | 1 | 12 | 12.9800 | 1.2849 | 9.83 | 10.10 | <.0001 | Tukey-Kramer | 0.0006 |
| treatment | 1 | 13 | 8.9967 | 0.9382 | 7.14 | 9.59 | <.0001 | Tukey-Kramer | 0.0008 |
| treatment | 1 | 14 | 3.6450 | 2.1925 | 6.7 | 1.66 | 0.1423 | Tukey-Kramer | 0.9644 |
| treatment | 1 | 15 | 8.5400 | 0.8732 | 5.61 | 9.78 | <.0001 | Tukey-Kramer | 0.0007 |
| treatment | 1 | 16 | 5.2607 | 1.3373 | 8.21 | 3.93 | 0.0041 | Tukey-Kramer | 0.1669 |
| treatment | 1 | 17 | 9.8333 | 0.9435 | 7.26 | 10.42 | <.0001 | Tukey-Kramer | 0.0004 |
| treatment | 1 | 18 | 4.4227 | 1.1653 | 8.97 | 3.80 | 0.0043 | Tukey-Kramer | 0.1939 |
| treatment | 1 | 19 | 6.4117 | 1.0543 | 9.21 | 6.08 | 0.0002 | Tukey-Kramer | 0.0168 |
| treatment | 1 | 20 | 0.8647 | 1.6992 | 6.52 | 0.51 | 0.6276 | Tukey-Kramer | 1.0000 |
| treatment | 1 | 21 | 6.4250 | 0.9934 | 8.28 | 6.47 | 0.0002 | Tukey-Kramer | 0.0115 |
| treatment | 1 | 22 | 1.1407 | 1.0128 | 8.3 | 1.13 | 0.2916 | Tukey-Kramer | 0.9993 |
| treatment | 1 | 23 | 5.9417 | 1.0440 | 9.08 | 5.69 | 0.0003 | Tukey-Kramer | 0.0250 |
| treatment | 1 | 24 | 2.6367 | 1.1859 | 8.93 | 2.22 | 0.0535 | Tukey-Kramer | 0.7908 |
| treatment | 2 | 3 | 15.5283 | 2.3982 | 5.27 | 6.48 | 0.0011 | Tukey-Kramer | 0.0114 |
| treatment | 2 | 4 | 21.3083 | 2.4962 | 6.11 | 8.54 | 0.0001 | Tukey-Kramer | 0.0018 |
| treatment | 2 | 5 | 19.4217 | 2.7195 | 7.9 | 7.14 | 0.0001 | Tukey-Kramer | 0.0061 |
| treatment | 2 | 6 | 10.7450 | 2.9008 | 9.01 | 3.70 | 0.0049 | Tukey-Kramer | 0.2139 |
| treatment | 2 | 7 | 35.6917 | 2.4213 | 5.47 | 14.74 | <.0001 | Tukey-Kramer | <.0001 |
| treatment | 2 | 8 | 7.7767 | 4.1798 | 8.86 | 1.86 | 0.0963 | Tukey-Kramer | 0.9213 |
| treatment | 2 | 9 | 37.0017 | 2.4194 | 5.45 | 15.29 | <.0001 | Tukey-Kramer | <.0001 |
| treatment | 2 | 10 | 34.8600 | 2.4021 | 5.3 | 14.51 | <.0001 | Tukey-Kramer | <.0001 |
| treatment | 2 | 11 | 36.2517 | 2.3672 | 5 | 15.31 | <.0001 | Tukey-Kramer | <.0001 |
| treatment | 2 | 12 | 30.1400 | 2.5562 | 6.62 | 11.79 | <.0001 | Tukey-Kramer | 0.0002 |

# Supporting Information

| | | | | | | | | | |
|---|---|---|---|---|---|---|---|---|---|
| treatment | 2 | 13 | 26.1567 | 2.4007 | 5.29 | 10.90 | <.0001 | Tukey-Kramer | 0.0003 |
| treatment | 2 | 14 | 20.8050 | 3.1129 | 9.76 | 6.68 | <.0001 | Tukey-Kramer | 0.0093 |
| treatment | 2 | 15 | 25.7000 | 2.3761 | 5.08 | 10.82 | 0.0001 | Tukey-Kramer | 0.0003 |
| treatment | 2 | 16 | 22.4207 | 2.5830 | 6.78 | 8.68 | <.0001 | Tukey-Kramer | 0.0016 |
| treatment | 2 | 17 | 26.9933 | 2.4028 | 5.31 | 11.23 | <.0001 | Tukey-Kramer | 0.0003 |
| treatment | 2 | 18 | 21.5827 | 2.4982 | 6.11 | 8.64 | 0.0001 | Tukey-Kramer | 0.0017 |
| treatment | 2 | 19 | 23.5717 | 2.4484 | 5.7 | 9.63 | <.0001 | Tukey-Kramer | 0.0008 |
| treatment | 2 | 20 | 18.0247 | 2.7876 | 8.1 | 6.47 | 0.0002 | Tukey-Kramer | 0.0115 |
| treatment | 2 | 21 | 23.5850 | 2.4228 | 5.48 | 9.73 | 0.0001 | Tukey-Kramer | 0.0007 |
| treatment | 2 | 22 | 18.3007 | 2.4309 | 5.54 | 7.53 | 0.0004 | Tukey-Kramer | 0.0043 |
| treatment | 2 | 23 | 23.1017 | 2.4440 | 5.66 | 9.45 | 0.0001 | Tukey-Kramer | 0.0009 |
| treatment | 2 | 24 | 19.7967 | 2.5079 | 6.19 | 7.89 | 0.0002 | Tukey-Kramer | 0.0031 |
| treatment | 3 | 4 | 5.7800 | 0.8826 | 7.25 | 6.55 | 0.0003 | Tukey-Kramer | 0.0106 |
| treatment | 3 | 5 | 3.8933 | 1.3942 | 5.83 | 2.79 | 0.0325 | Tukey-Kramer | 0.5268 |
| treatment | 3 | 6 | -4.7833 | 1.7212 | 5.53 | -2.78 | 0.0349 | Tukey-Kramer | 0.5330 |
| treatment | 3 | 7 | 20.1633 | 0.6410 | 9.31 | 31.45 | <.0001 | Tukey-Kramer | <.0001 |
| treatment | 3 | 8 | -7.7517 | 3.4668 | 5.13 | -2.24 | 0.0743 | Tukey-Kramer | 0.7854 |
| treatment | 3 | 9 | 21.4733 | 0.6339 | 9.39 | 33.87 | <.0001 | Tukey-Kramer | <.0001 |
| treatment | 3 | 10 | 19.3317 | 0.5644 | 9.96 | 34.25 | <.0001 | Tukey-Kramer | <.0001 |
| treatment | 3 | 11 | 20.7233 | 0.3895 | 5.13 | 53.21 | <.0001 | Tukey-Kramer | <.0001 |
| treatment | 3 | 12 | 14.6117 | 1.0404 | 6.56 | 14.04 | <.0001 | Tukey-Kramer | <.0001 |
| treatment | 3 | 13 | 10.6283 | 0.5582 | 9.98 | 19.04 | <.0001 | Tukey-Kramer | <.0001 |
| treatment | 3 | 14 | 5.2767 | 2.0588 | 5.37 | 2.56 | 0.0473 | Tukey-Kramer | 0.6338 |
| treatment | 3 | 15 | 10.1717 | 0.4404 | 7.72 | 23.10 | <.0001 | Tukey-Kramer | <.0001 |
| treatment | 3 | 16 | 6.8923 | 1.1045 | 5.12 | 6.24 | 0.0014 | Tukey-Kramer | 0.0143 |
| treatment | 3 | 17 | 11.4650 | 0.5672 | 9.95 | 20.21 | <.0001 | Tukey-Kramer | <.0001 |
| treatment | 3 | 18 | 6.0543 | 0.8885 | 5.84 | 6.81 | 0.0006 | Tukey-Kramer | 0.0082 |
| treatment | 3 | 19 | 8.0433 | 0.7369 | 8.32 | 10.91 | <.0001 | Tukey-Kramer | 0.0003 |
| treatment | 3 | 20 | 2.4963 | 1.5228 | 4.55 | 1.64 | 0.1678 | Tukey-Kramer | 0.9681 |
| treatment | 3 | 21 | 8.0567 | 0.6467 | 9.25 | 12.46 | <.0001 | Tukey-Kramer | 0.0001 |
| treatment | 3 | 22 | 2.7723 | 0.6762 | 7.43 | 4.10 | 0.0040 | Tukey-Kramer | 0.1391 |
| treatment | 3 | 23 | 7.5733 | 0.7221 | 8.47 | 10.49 | <.0001 | Tukey-Kramer | 0.0004 |
| treatment | 3 | 24 | 4.2683 | 0.9153 | 5.71 | 4.66 | 0.0039 | Tukey-Kramer | 0.0749 |

# Supporting Information

| | | | | | | | | | |
|---|---|---|---|---|---|---|---|---|---|
| treatment | 4 | 5 | -1.8867 | 1.5567 | 8.12 | -1.21 | 0.2596 | Tukey-Kramer | 0.9983 |
| treatment | 4 | 6 | -10.5633 | 1.8553 | 7.13 | -5.69 | 0.0007 | Tukey-Kramer | 0.0250 |
| treatment | 4 | 7 | 14.3833 | 0.9437 | 8.54 | 15.24 | <.0001 | Tukey-Kramer | <.0001 |
| treatment | 4 | 8 | -13.5317 | 3.5353 | 5.53 | -3.83 | 0.0101 | Tukey-Kramer | 0.1873 |
| treatment | 4 | 9 | 15.6933 | 0.9389 | 8.45 | 16.72 | <.0001 | Tukey-Kramer | <.0001 |
| treatment | 4 | 10 | 13.5517 | 0.8934 | 7.5 | 15.17 | <.0001 | Tukey-Kramer | <.0001 |
| treatment | 4 | 11 | 14.9433 | 0.7945 | 5.03 | 18.81 | <.0001 | Tukey-Kramer | <.0001 |
| treatment | 4 | 12 | 8.8317 | 1.2498 | 9.64 | 7.07 | <.0001 | Tukey-Kramer | 0.0065 |
| treatment | 4 | 13 | 4.8483 | 0.8895 | 7.41 | 5.45 | 0.0008 | Tukey-Kramer | 0.0321 |
| treatment | 4 | 14 | -0.5033 | 2.1721 | 6.5 | -0.23 | 0.8239 | Tukey-Kramer | 1.0000 |
| treatment | 4 | 15 | 4.3917 | 0.8207 | 5.7 | 5.35 | 0.0020 | Tukey-Kramer | 0.0357 |
| treatment | 4 | 16 | 1.1123 | 1.3037 | 7.9 | 0.85 | 0.4186 | Tukey-Kramer | 1.0000 |
| treatment | 4 | 17 | 5.6850 | 0.8952 | 7.54 | 6.35 | 0.0003 | Tukey-Kramer | 0.0129 |
| treatment | 4 | 18 | 0.2743 | 1.1265 | 8.87 | 0.24 | 0.8131 | Tukey-Kramer | 1.0000 |
| treatment | 4 | 19 | 2.2633 | 1.0113 | 9.49 | 2.24 | 0.0505 | Tukey-Kramer | 0.7844 |
| treatment | 4 | 20 | -3.2837 | 1.6728 | 6.24 | -1.96 | 0.0955 | Tukey-Kramer | 0.8906 |
| treatment | 4 | 21 | 2.2767 | 0.9475 | 8.61 | 2.40 | 0.0409 | Tukey-Kramer | 0.7097 |
| treatment | 4 | 22 | -3.0077 | 0.9679 | 8.53 | -3.11 | 0.0134 | Tukey-Kramer | 0.3951 |
| treatment | 4 | 23 | 1.7933 | 1.0005 | 9.38 | 1.79 | 0.1053 | Tukey-Kramer | 0.9385 |
| treatment | 4 | 24 | -1.5117 | 1.1478 | 8.78 | -1.32 | 0.2211 | Tukey-Kramer | 0.9957 |
| treatment | 5 | 6 | -8.6767 | 2.1464 | 9.53 | -4.04 | 0.0026 | Tukey-Kramer | 0.1482 |
| treatment | 5 | 7 | 16.2700 | 1.4336 | 6.43 | 11.35 | <.0001 | Tukey-Kramer | 0.0002 |
| treatment | 5 | 8 | -11.6450 | 3.6964 | 6.48 | -3.15 | 0.0179 | Tukey-Kramer | 0.3789 |
| treatment | 5 | 9 | 17.5800 | 1.4304 | 6.38 | 12.29 | <.0001 | Tukey-Kramer | 0.0001 |
| treatment | 5 | 10 | 15.4383 | 1.4010 | 5.93 | 11.02 | <.0001 | Tukey-Kramer | 0.0003 |
| treatment | 5 | 11 | 16.8300 | 1.3401 | 5.01 | 12.56 | <.0001 | Tukey-Kramer | 0.0001 |
| treatment | 5 | 12 | 10.7183 | 1.6513 | 9.09 | 6.49 | 0.0001 | Tukey-Kramer | 0.0112 |
| treatment | 5 | 13 | 6.7350 | 1.3985 | 5.9 | 4.82 | 0.0031 | Tukey-Kramer | 0.0634 |
| treatment | 5 | 14 | 1.3833 | 2.4255 | 8.68 | 0.57 | 0.5829 | Tukey-Kramer | 1.0000 |
| treatment | 5 | 15 | 6.2783 | 1.3558 | 5.25 | 4.63 | 0.0050 | Tukey-Kramer | 0.0777 |
| treatment | 5 | 16 | 2.9990 | 1.6924 | 8.82 | 1.77 | 0.1108 | Tukey-Kramer | 0.9432 |
| treatment | 5 | 17 | 7.5717 | 1.4021 | 5.95 | 5.40 | 0.0017 | Tukey-Kramer | 0.0339 |

# Supporting Information

| | | | | | | | | | |
|---|---|---|---|---|---|---|---|---|---|
| treatment | 5 | 18 | 2.1610 | 1.5600 | 7.94 | 1.39 | 0.2037 | Tukey-Kramer | 0.9928 |
| treatment | 5 | 19 | 4.1500 | 1.4790 | 7.09 | 2.81 | 0.0259 | Tukey-Kramer | 0.5208 |
| treatment | 5 | 20 | -1.3970 | 1.9907 | 8.63 | -0.70 | 0.5013 | Tukey-Kramer | 1.0000 |
| treatment | 5 | 21 | 4.1633 | 1.4362 | 6.46 | 2.90 | 0.0252 | Tukey-Kramer | 0.4799 |
| treatment | 5 | 22 | -1.1210 | 1.4497 | 6.62 | -0.77 | 0.4661 | Tukey-Kramer | 1.0000 |
| treatment | 5 | 23 | 3.6800 | 1.4716 | 6.99 | 2.50 | 0.0410 | Tukey-Kramer | 0.6634 |
| treatment | 5 | 24 | 0.3750 | 1.5755 | 8.08 | 0.24 | 0.8178 | Tukey-Kramer | 1.0000 |
| treatment | 6 | 7 | 24.9467 | 1.7533 | 5.92 | 14.23 | <.0001 | Tukey-Kramer | <.0001 |
| treatment | 6 | 8 | -2.9683 | 3.8317 | 7.24 | -0.77 | 0.4631 | Tukey-Kramer | 1.0000 |
| treatment | 6 | 9 | 26.2567 | 1.7507 | 5.89 | 15.00 | <.0001 | Tukey-Kramer | <.0001 |
| treatment | 6 | 10 | 24.1150 | 1.7267 | 5.6 | 13.97 | <.0001 | Tukey-Kramer | <.0001 |
| treatment | 6 | 11 | 25.5067 | 1.6777 | 5.01 | 15.20 | <.0001 | Tukey-Kramer | <.0001 |
| treatment | 6 | 12 | 19.3950 | 1.9354 | 7.99 | 10.02 | <.0001 | Tukey-Kramer | 0.0006 |
| treatment | 6 | 13 | 15.4117 | 1.7247 | 5.57 | 8.94 | 0.0002 | Tukey-Kramer | 0.0013 |
| treatment | 6 | 14 | 10.0600 | 2.6271 | 9.67 | 3.83 | 0.0035 | Tukey-Kramer | 0.1869 |
| treatment | 6 | 15 | 14.9550 | 1.6903 | 5.16 | 8.85 | 0.0003 | Tukey-Kramer | 0.0014 |
| treatment | 6 | 16 | 11.6757 | 1.9705 | 8.07 | 5.93 | 0.0003 | Tukey-Kramer | 0.0197 |
| treatment | 6 | 17 | 16.2483 | 1.7277 | 5.61 | 9.40 | 0.0001 | Tukey-Kramer | 0.0009 |
| treatment | 6 | 18 | 10.8377 | 1.8581 | 7.08 | 5.83 | 0.0006 | Tukey-Kramer | 0.0216 |
| treatment | 6 | 19 | 12.8267 | 1.7906 | 6.37 | 7.16 | 0.0003 | Tukey-Kramer | 0.0060 |
| treatment | 6 | 20 | 7.2797 | 2.2320 | 9 | 3.26 | 0.0098 | Tukey-Kramer | 0.3394 |
| treatment | 6 | 21 | 12.8400 | 1.7554 | 5.95 | 7.31 | 0.0003 | Tukey-Kramer | 0.0052 |
| treatment | 6 | 22 | 7.5557 | 1.7665 | 6.06 | 4.28 | 0.0051 | Tukey-Kramer | 0.1145 |
| treatment | 6 | 23 | 12.3567 | 1.7845 | 6.3 | 6.92 | 0.0004 | Tukey-Kramer | 0.0074 |
| treatment | 6 | 24 | 9.0517 | 1.8711 | 7.21 | 4.84 | 0.0017 | Tukey-Kramer | 0.0619 |
| treatment | 7 | 8 | -27.9150 | 3.4828 | 5.22 | -8.01 | 0.0004 | Tukey-Kramer | 0.0028 |
| treatment | 7 | 9 | 1.3100 | 0.7165 | 10 | 1.83 | 0.0974 | Tukey-Kramer | 0.9297 |
| treatment | 7 | 10 | -0.8317 | 0.6557 | 9.56 | -1.27 | 0.2347 | Tukey-Kramer | 0.9971 |
| treatment | 7 | 11 | 0.5600 | 0.5130 | 5.08 | 1.09 | 0.3241 | Tukey-Kramer | 0.9995 |
| treatment | 7 | 12 | -5.5517 | 1.0927 | 7.6 | -5.08 | 0.0011 | Tukey-Kramer | 0.0476 |
| treatment | 7 | 13 | -9.5350 | 0.6504 | 9.48 | -14.66 | <.0001 | Tukey-Kramer | <.0001 |
| treatment | 7 | 14 | -14.8867 | 2.0857 | 5.64 | -7.14 | 0.0005 | Tukey-Kramer | 0.0061 |

# Supporting Information

| | | | | | | | | | |
|---|---|---|---|---|---|---|---|---|---|
| treatment | 7 | 15 | -9.9917 | 0.5526 | 6.65 | -18.08 | <.0001 | Tukey-Kramer | <.0001 |
| treatment | 7 | 16 | -13.2710 | 1.1539 | 5.91 | -11.50 | <.0001 | Tukey-Kramer | 0.0002 |
| treatment | 7 | 17 | -8.6983 | 0.6582 | 9.59 | -13.22 | <.0001 | Tukey-Kramer | <.0001 |
| treatment | 7 | 18 | -14.1090 | 0.9491 | 7 | -14.87 | <.0001 | Tukey-Kramer | <.0001 |
| treatment | 7 | 19 | -12.1200 | 0.8090 | 9.61 | -14.98 | <.0001 | Tukey-Kramer | <.0001 |
| treatment | 7 | 20 | -17.6670 | 1.5589 | 4.96 | -11.33 | <.0001 | Tukey-Kramer | 0.0002 |
| treatment | 7 | 21 | -12.1067 | 0.7278 | 10 | -16.63 | <.0001 | Tukey-Kramer | <.0001 |
| treatment | 7 | 22 | -17.3910 | 0.7541 | 8.67 | -23.06 | <.0001 | Tukey-Kramer | <.0001 |
| treatment | 7 | 23 | -12.5900 | 0.7956 | 9.7 | -15.83 | <.0001 | Tukey-Kramer | <.0001 |
| treatment | 7 | 24 | -15.8950 | 0.9743 | 6.83 | -16.31 | <.0001 | Tukey-Kramer | <.0001 |
| treatment | 8 | 9 | 29.2250 | 3.4816 | 5.21 | 8.39 | 0.0003 | Tukey-Kramer | 0.0020 |
| treatment | 8 | 10 | 27.0833 | 3.4696 | 5.14 | 7.81 | 0.0005 | Tukey-Kramer | 0.0034 |
| treatment | 8 | 11 | 28.4750 | 3.4454 | 5 | 8.26 | 0.0004 | Tukey-Kramer | 0.0023 |
| treatment | 8 | 12 | 22.3633 | 3.5780 | 5.78 | 6.25 | 0.0009 | Tukey-Kramer | 0.0142 |
| treatment | 8 | 13 | 18.3800 | 3.4686 | 5.14 | 5.30 | 0.0029 | Tukey-Kramer | 0.0377 |
| treatment | 8 | 14 | 13.0283 | 3.9947 | 8.08 | 3.26 | 0.0113 | Tukey-Kramer | 0.3395 |
| treatment | 8 | 15 | 17.9233 | 3.4516 | 5.04 | 5.19 | 0.0034 | Tukey-Kramer | 0.0422 |
| treatment | 8 | 16 | 14.6440 | 3.5971 | 5.88 | 4.07 | 0.0068 | Tukey-Kramer | 0.1436 |
| treatment | 8 | 17 | 19.2167 | 3.4700 | 5.14 | 5.54 | 0.0024 | Tukey-Kramer | 0.0293 |
| treatment | 8 | 18 | 13.8060 | 3.5368 | 5.53 | 3.90 | 0.0093 | Tukey-Kramer | 0.1724 |
| treatment | 8 | 19 | 15.7950 | 3.5018 | 5.33 | 4.51 | 0.0054 | Tukey-Kramer | 0.0886 |
| treatment | 8 | 20 | 10.2480 | 3.7467 | 6.71 | 2.74 | 0.0303 | Tukey-Kramer | 0.5530 |
| treatment | 8 | 21 | 15.8083 | 3.4839 | 5.23 | 4.54 | 0.0055 | Tukey-Kramer | 0.0860 |
| treatment | 8 | 22 | 10.5240 | 3.4895 | 5.26 | 3.02 | 0.0277 | Tukey-Kramer | 0.4310 |
| treatment | 8 | 23 | 15.3250 | 3.4987 | 5.31 | 4.38 | 0.0062 | Tukey-Kramer | 0.1022 |
| treatment | 8 | 24 | 12.0200 | 3.5436 | 5.57 | 3.39 | 0.0164 | Tukey-Kramer | 0.2972 |
| treatment | 9 | 10 | -2.1417 | 0.6488 | 9.62 | -3.30 | 0.0084 | Tukey-Kramer | 0.3262 |
| treatment | 9 | 11 | -0.7500 | 0.5041 | 5.08 | -1.49 | 0.1961 | Tukey-Kramer | 0.9859 |
| treatment | 9 | 12 | -6.8617 | 1.0886 | 7.52 | -6.30 | 0.0003 | Tukey-Kramer | 0.0135 |
| treatment | 9 | 13 | -10.8450 | 0.6435 | 9.55 | -16.85 | <.0001 | Tukey-Kramer | <.0001 |
| treatment | 9 | 14 | -16.1967 | 2.0835 | 5.61 | -7.77 | 0.0003 | Tukey-Kramer | 0.0035 |
| treatment | 9 | 15 | -11.3017 | 0.5444 | 6.7 | -20.76 | <.0001 | Tukey-Kramer | <.0001 |

# Supporting Information

| | | | | | | | | | |
|---|---|---|---|---|---|---|---|---|---|
| treatment | 9 | 16 | -14.5810 | 1.1499 | 5.85 | -12.68 | <.0001 | Tukey-Kramer | 0.0001 |
| treatment | 9 | 17 | -10.0083 | 0.6513 | 9.66 | -15.37 | <.0001 | Tukey-Kramer | <.0001 |
| treatment | 9 | 18 | -15.4190 | 0.9443 | 6.92 | -16.33 | <.0001 | Tukey-Kramer | <.0001 |
| treatment | 9 | 19 | -13.4300 | 0.8034 | 9.54 | -16.72 | <.0001 | Tukey-Kramer | <.0001 |
| treatment | 9 | 20 | -18.9770 | 1.5560 | 4.93 | -12.20 | <.0001 | Tukey-Kramer | 0.0001 |
| treatment | 9 | 21 | -13.4167 | 0.7216 | 9.99 | -18.59 | <.0001 | Tukey-Kramer | <.0001 |
| treatment | 9 | 22 | -18.7010 | 0.7481 | 8.62 | -25.00 | <.0001 | Tukey-Kramer | <.0001 |
| treatment | 9 | 23 | -13.9000 | 0.7899 | 9.65 | -17.60 | <.0001 | Tukey-Kramer | <.0001 |
| treatment | 9 | 24 | -17.2050 | 0.9697 | 6.74 | -17.74 | <.0001 | Tukey-Kramer | <.0001 |
| treatment | 10 | 11 | 1.3917 | 0.4132 | 5.12 | 3.37 | 0.0192 | Tukey-Kramer | 0.3047 |
| treatment | 10 | 12 | -4.7200 | 1.0496 | 6.75 | -4.50 | 0.0031 | Tukey-Kramer | 0.0899 |
| treatment | 10 | 13 | -8.7033 | 0.5750 | 10 | -15.14 | <.0001 | Tukey-Kramer | <.0001 |
| treatment | 10 | 14 | -14.0550 | 2.0634 | 5.41 | -6.81 | 0.0008 | Tukey-Kramer | 0.0083 |
| treatment | 10 | 15 | -9.1600 | 0.4615 | 7.45 | -19.85 | <.0001 | Tukey-Kramer | <.0001 |
| treatment | 10 | 16 | -12.4393 | 1.1131 | 5.26 | -11.18 | <.0001 | Tukey-Kramer | 0.0003 |
| treatment | 10 | 17 | -7.8667 | 0.5838 | 10 | -13.48 | <.0001 | Tukey-Kramer | <.0001 |
| treatment | 10 | 18 | -13.2773 | 0.8991 | 6.05 | -14.77 | <.0001 | Tukey-Kramer | <.0001 |
| treatment | 10 | 19 | -11.2883 | 0.7498 | 8.62 | -15.06 | <.0001 | Tukey-Kramer | <.0001 |
| treatment | 10 | 20 | -16.8353 | 1.5290 | 4.62 | -11.01 | 0.0002 | Tukey-Kramer | 0.0003 |
| treatment | 10 | 21 | -11.2750 | 0.6613 | 9.51 | -17.05 | <.0001 | Tukey-Kramer | <.0001 |
| treatment | 10 | 22 | -16.5593 | 0.6901 | 7.73 | -23.99 | <.0001 | Tukey-Kramer | <.0001 |
| treatment | 10 | 23 | -11.7583 | 0.7352 | 8.76 | -15.99 | <.0001 | Tukey-Kramer | <.0001 |
| treatment | 10 | 24 | -15.0633 | 0.9257 | 5.92 | -16.27 | <.0001 | Tukey-Kramer | <.0001 |
| treatment | 11 | 12 | -6.1117 | 0.9668 | 5.02 | -6.32 | 0.0014 | Tukey-Kramer | 0.0132 |
| treatment | 11 | 13 | -10.0950 | 0.4048 | 5.12 | -24.94 | <.0001 | Tukey-Kramer | <.0001 |
| treatment | 11 | 14 | -15.4467 | 2.0226 | 5 | -7.64 | 0.0006 | Tukey-Kramer | 0.0039 |
| treatment | 11 | 15 | -10.5517 | 0.2149 | 5.45 | -49.10 | <.0001 | Tukey-Kramer | <.0001 |
| treatment | 11 | 16 | -13.8310 | 1.0355 | 4.01 | -13.36 | 0.0002 | Tukey-Kramer | <.0001 |
| treatment | 11 | 17 | -9.2583 | 0.4171 | 5.12 | -22.20 | <.0001 | Tukey-Kramer | <.0001 |
| treatment | 11 | 18 | -14.6690 | 0.8010 | 4.02 | -18.31 | <.0001 | Tukey-Kramer | <.0001 |
| treatment | 11 | 19 | -12.6800 | 0.6288 | 5.05 | -20.17 | <.0001 | Tukey-Kramer | <.0001 |
| treatment | 11 | 20 | -18.2270 | 1.4734 | 4.01 | -12.37 | 0.0002 | Tukey-Kramer | 0.0001 |

# Supporting Information

| | | | | | | | | | |
|---|---|---|---|---|---|---|---|---|---|
| treatment | 11 | 21 | -12.6667 | 0.5201 | 5.07 | -24.36 | <.0001 | Tukey-Kramer | <.0001 |
| treatment | 11 | 22 | -17.9510 | 0.5563 | 4.05 | -32.27 | <.0001 | Tukey-Kramer | <.0001 |
| treatment | 11 | 23 | -13.1500 | 0.6113 | 5.05 | -21.51 | <.0001 | Tukey-Kramer | <.0001 |
| treatment | 11 | 24 | -16.4550 | 0.8307 | 4.02 | -19.81 | <.0001 | Tukey-Kramer | <.0001 |
| treatment | 12 | 13 | -3.9833 | 1.0463 | 6.68 | -3.81 | 0.0073 | Tukey-Kramer | 0.1914 |
| treatment | 12 | 14 | -9.3350 | 2.2409 | 7.17 | -4.17 | 0.0040 | Tukey-Kramer | 0.1294 |
| treatment | 12 | 15 | -4.4400 | 0.9884 | 5.47 | -4.49 | 0.0052 | Tukey-Kramer | 0.0904 |
| treatment | 12 | 16 | -7.7193 | 1.4153 | 8.71 | -5.45 | 0.0005 | Tukey-Kramer | 0.0320 |
| treatment | 12 | 17 | -3.1467 | 1.0511 | 6.78 | -2.99 | 0.0209 | Tukey-Kramer | 0.4400 |
| treatment | 12 | 18 | -8.5573 | 1.2540 | 8.95 | -6.82 | <.0001 | Tukey-Kramer | 0.0082 |
| treatment | 12 | 19 | -6.5683 | 1.1516 | 8.58 | -5.70 | 0.0003 | Tukey-Kramer | 0.0247 |
| treatment | 12 | 20 | -12.1153 | 1.7612 | 7.13 | -6.88 | 0.0002 | Tukey-Kramer | 0.0077 |
| treatment | 12 | 21 | -6.5550 | 1.0960 | 7.66 | -5.98 | 0.0004 | Tukey-Kramer | 0.0186 |
| treatment | 12 | 22 | -11.8393 | 1.1137 | 7.78 | -10.63 | <.0001 | Tukey-Kramer | 0.0004 |
| treatment | 12 | 23 | -7.0383 | 1.1421 | 8.44 | -6.16 | 0.0002 | Tukey-Kramer | 0.0155 |
| treatment | 12 | 24 | -10.3433 | 1.2731 | 8.99 | -8.12 | <.0001 | Tukey-Kramer | 0.0026 |
| treatment | 13 | 14 | -5.3517 | 2.0617 | 5.4 | -2.60 | 0.0451 | Tukey-Kramer | 0.6183 |
| treatment | 13 | 15 | -0.4567 | 0.4540 | 7.54 | -1.01 | 0.3456 | Tukey-Kramer | 0.9998 |
| treatment | 13 | 16 | -3.7360 | 1.1100 | 5.21 | -3.37 | 0.0188 | Tukey-Kramer | 0.3054 |
| treatment | 13 | 17 | 0.8367 | 0.5778 | 9.99 | 1.45 | 0.1783 | Tukey-Kramer | 0.9890 |
| treatment | 13 | 18 | -4.5740 | 0.8953 | 5.97 | -5.11 | 0.0022 | Tukey-Kramer | 0.0462 |
| treatment | 13 | 19 | -2.5850 | 0.7451 | 8.52 | -3.47 | 0.0077 | Tukey-Kramer | 0.2744 |
| treatment | 13 | 20 | -8.1320 | 1.5267 | 4.6 | -5.33 | 0.0040 | Tukey-Kramer | 0.0366 |
| treatment | 13 | 21 | -2.5717 | 0.6560 | 9.42 | -3.92 | 0.0032 | Tukey-Kramer | 0.1694 |
| treatment | 13 | 22 | -7.8560 | 0.6851 | 7.63 | -11.47 | <.0001 | Tukey-Kramer | 0.0002 |
| treatment | 13 | 23 | -3.0550 | 0.7305 | 8.66 | -4.18 | 0.0026 | Tukey-Kramer | 0.1271 |
| treatment | 13 | 24 | -6.3600 | 0.9219 | 5.84 | -6.90 | 0.0005 | Tukey-Kramer | 0.0076 |
| treatment | 14 | 15 | 4.8950 | 2.0330 | 5.11 | 2.41 | 0.0600 | Tukey-Kramer | 0.7073 |
| treatment | 14 | 16 | 1.6157 | 2.2713 | 7.33 | 0.71 | 0.4989 | Tukey-Kramer | 1.0000 |
| treatment | 14 | 17 | 6.1883 | 2.0642 | 5.42 | 3.00 | 0.0273 | Tukey-Kramer | 0.4383 |
| treatment | 14 | 18 | 0.7777 | 2.1745 | 6.49 | 0.36 | 0.7320 | Tukey-Kramer | 1.0000 |

| | | | | | | | | | |
|---|---|---|---|---|---|---|---|---|---|
| treatment | 14 | 19 | 2.7667 | 2.1171 | 5.95 | 1.31 | 0.2395 | Tukey-Kramer | 0.9960 |
| treatment | 14 | 20 | -2.7803 | 2.5016 | 8.66 | -1.11 | 0.2963 | Tukey-Kramer | 0.9994 |
| treatment | 14 | 21 | 2.7800 | 2.0874 | 5.65 | 1.33 | 0.2341 | Tukey-Kramer | 0.9952 |
| treatment | 14 | 22 | -2.5043 | 2.0967 | 5.74 | -1.19 | 0.2793 | Tukey-Kramer | 0.9985 |
| treatment | 14 | 23 | 2.2967 | 2.1120 | 5.9 | 1.09 | 0.3192 | Tukey-Kramer | 0.9995 |
| treatment | 14 | 24 | -1.0083 | 2.1856 | 6.59 | -0.46 | 0.6594 | Tukey-Kramer | 1.0000 |
| treatment | 15 | 16 | -3.2793 | 1.0557 | 4.33 | -3.11 | 0.0323 | Tukey-Kramer | 0.3954 |
| treatment | 15 | 17 | 1.2933 | 0.4650 | 7.41 | 2.78 | 0.0257 | Tukey-Kramer | 0.5318 |
| treatment | 15 | 18 | -4.1173 | 0.8269 | 4.55 | -4.98 | 0.0054 | Tukey-Kramer | 0.0532 |
| treatment | 15 | 19 | -2.1283 | 0.6615 | 6.11 | -3.22 | 0.0177 | Tukey-Kramer | 0.3546 |
| treatment | 15 | 20 | -7.6753 | 1.4877 | 4.16 | -5.16 | 0.0060 | Tukey-Kramer | 0.0438 |
| treatment | 15 | 21 | -2.1150 | 0.5592 | 6.6 | -3.78 | 0.0077 | Tukey-Kramer | 0.1967 |
| treatment | 15 | 22 | -7.3993 | 0.5931 | 5.15 | -12.48 | <.0001 | Tukey-Kramer | 0.0001 |
| treatment | 15 | 23 | -2.5983 | 0.6449 | 6.17 | -4.03 | 0.0065 | Tukey-Kramer | 0.1504 |
| treatment | 15 | 24 | -5.9033 | 0.8557 | 4.52 | -6.90 | 0.0015 | Tukey-Kramer | 0.0076 |
| treatment | 16 | 17 | 4.5727 | 1.1146 | 5.28 | 4.10 | 0.0083 | Tukey-Kramer | 0.1387 |
| treatment | 16 | 18 | -0.8380 | 1.3076 | 7.52 | -0.64 | 0.5406 | Tukey-Kramer | 1.0000 |
| treatment | 16 | 19 | 1.1510 | 1.2098 | 6.75 | 0.95 | 0.3742 | Tukey-Kramer | 0.9999 |
| treatment | 16 | 20 | -4.3960 | 1.7998 | 7.17 | -2.44 | 0.0438 | Tukey-Kramer | 0.6910 |
| treatment | 16 | 21 | 1.1643 | 1.1570 | 5.96 | 1.01 | 0.3534 | Tukey-Kramer | 0.9998 |
| treatment | 16 | 22 | -4.1200 | 1.1738 | 6.12 | -3.51 | 0.0123 | Tukey-Kramer | 0.2629 |
| treatment | 16 | 23 | 0.6810 | 1.2008 | 6.62 | 0.57 | 0.5893 | Tukey-Kramer | 1.0000 |
| treatment | 16 | 24 | -2.6240 | 1.3260 | 7.64 | -1.98 | 0.0849 | Tukey-Kramer | 0.8854 |
| treatment | 17 | 18 | -5.4107 | 0.9009 | 6.09 | -6.01 | 0.0009 | Tukey-Kramer | 0.0181 |
| treatment | 17 | 19 | -3.4217 | 0.7519 | 8.67 | -4.55 | 0.0015 | Tukey-Kramer | 0.0848 |
| treatment | 17 | 20 | -8.9687 | 1.5301 | 4.64 | -5.86 | 0.0026 | Tukey-Kramer | 0.0210 |
| treatment | 17 | 21 | -3.4083 | 0.6637 | 9.54 | -5.14 | 0.0005 | Tukey-Kramer | 0.0449 |
| treatment | 17 | 22 | -8.6927 | 0.6925 | 7.78 | -12.55 | <.0001 | Tukey-Kramer | 0.0001 |
| treatment | 17 | 23 | -3.8917 | 0.7374 | 8.81 | -5.28 | 0.0005 | Tukey-Kramer | 0.0386 |
| treatment | 17 | 24 | -7.1967 | 0.9274 | 5.95 | -7.76 | 0.0003 | Tukey-Kramer | 0.0035 |
| treatment | 18 | 19 | 1.9890 | 1.0164 | 8.01 | 1.96 | 0.0860 | Tukey-Kramer | 0.8926 |
| treatment | 18 | 20 | -3.5580 | 1.6759 | 6.17 | -2.12 | 0.0767 | Tukey-Kramer | 0.8325 |

# Supporting Information

| | | | | | | | | | |
|---|---|---|---|---|---|---|---|---|---|
| treatment | 18 | 21 | 2.0023 | 0.9530 | 7.07 | 2.10 | 0.0734 | Tukey-Kramer | 0.8411 |
| treatment | 18 | 22 | -3.2820 | 0.9732 | 7.12 | -3.37 | 0.0116 | Tukey-Kramer | 0.3033 |
| treatment | 18 | 23 | 1.5190 | 1.0057 | 7.87 | 1.51 | 0.1700 | Tukey-Kramer | 0.9839 |
| treatment | 18 | 24 | -1.7860 | 1.1523 | 7.99 | -1.55 | 0.1598 | Tukey-Kramer | 0.9798 |
| treatment | 19 | 20 | -5.5470 | 1.6008 | 5.44 | -3.47 | 0.0157 | Tukey-Kramer | 0.2755 |
| treatment | 19 | 21 | 0.01333 | 0.8135 | 9.66 | 0.02 | 0.9873 | Tukey-Kramer | 1.0000 |
| treatment | 19 | 22 | -5.2710 | 0.8372 | 9 | -6.30 | 0.0001 | Tukey-Kramer | 0.0136 |
| treatment | 19 | 23 | -0.4700 | 0.8747 | 9.99 | -0.54 | 0.6028 | Tukey-Kramer | 1.0000 |
| treatment | 19 | 24 | -3.7750 | 1.0399 | 7.83 | -3.63 | 0.0069 | Tukey-Kramer | 0.2316 |
| treatment | 20 | 21 | 5.5603 | 1.5613 | 4.99 | 3.56 | 0.0162 | Tukey-Kramer | 0.2491 |
| treatment | 20 | 22 | 0.2760 | 1.5737 | 5.11 | 0.18 | 0.8675 | Tukey-Kramer | 1.0000 |
| treatment | 20 | 23 | 5.0770 | 1.5940 | 5.36 | 3.19 | 0.0221 | Tukey-Kramer | 0.3662 |
| treatment | 20 | 24 | 1.7720 | 1.6903 | 6.31 | 1.05 | 0.3330 | Tukey-Kramer | 0.9997 |
| treatment | 21 | 22 | -5.2843 | 0.7590 | 8.72 | -6.96 | <.0001 | Tukey-Kramer | 0.0072 |
| treatment | 21 | 23 | -0.4833 | 0.8001 | 9.75 | -0.60 | 0.5596 | Tukey-Kramer | 1.0000 |
| treatment | 21 | 24 | -3.7883 | 0.9780 | 6.89 | -3.87 | 0.0063 | Tukey-Kramer | 0.1782 |
| treatment | 22 | 23 | 4.8010 | 0.8242 | 9 | 5.83 | 0.0003 | Tukey-Kramer | 0.0218 |
| treatment | 22 | 24 | 1.4960 | 0.9978 | 6.98 | 1.50 | 0.1776 | Tukey-Kramer | 0.9849 |
| treatment | 23 | 24 | -3.3050 | 1.0295 | 7.69 | -3.21 | 0.0131 | Tukey-Kramer | 0.3572 |
